# Evidence Supporting Oral Hygiene Management by Owners through a Genetic Analysis of Dental Plaque Bacteria in Dogs

**DOI:** 10.3390/vetsci11020096

**Published:** 2024-02-19

**Authors:** Jeong suk Yu, Minhee Kim, Il-Hoon Cho, Yu-Min Sim, Young Sun Hwang

**Affiliations:** 1 Yeah Dental Animal Clinic, Seolleung-ro 126-6, Gangnam-Gu, Seoul 06092, Republic of Korea; 2 Department of Physical Therapy, College of Health Science, Eulji University, Seongnam 13135, Republic of Korea; 3 Department of Biomedical Laboratory Science, College of Health Science, Eulji University, Seongnam 13135, Republic of Korea; 4 Department of Dental Hygiene, College of Health Science, Eulji University, Seongnam 13135, Republic of Korea

**Keywords:** canine, dental hygiene, polymerase chain reaction, metagenome amplicon sequencing, *Streptococcus mutans*

## Abstract

**Simple Summary:**

With the increasing number of households owning dogs and the verified human-to-dog oral bacterial transmission, there is a rising concern regarding the oral hygiene of dogs. Our study focused on conducting a genetic analysis of dental plaque bacteria in dogs and investigating the oral hygiene practices of owners for their dogs. The results highlight the urgent need for owners to improve oral care practices for their dogs.

**Abstract:**

With the increase in the number of households raising dogs and the reports of human-to-dog transmission of oral bacteria, concerns about dogs’ oral health and the need for oral hygiene management are increasing. In this study, the owners’ perceptions about their dogs’ oral health and the frequency of oral hygiene were determined along with the analysis of dog dental plaque bacteria through metagenomic amplicon sequencing so as to support the need for oral hygiene management for dogs. Although the perception of 63.2% of the owners about their dogs’ oral health was consistent with the veterinarian’s diagnosis, the owners’ oral hygiene practices regarding their dogs were very poor. The calculi index (CI) and gingiva index (GI) were lower in dogs who had their teeth brushed more than once a week (57.89%) than in dogs brushed less than once a month (42.10%); however, the difference was nonsignificant (CI: *p* = 0.479, GI: *p* = 0.840). Genomic DNA was extracted from dental plaque bacteria removed during dog teeth scaling, and metagenomic amplicons were sequenced. The 16S amplicons of 73 species were identified from among the plaque bacteria of the dogs. These amplicons were of oral disease-causing bacteria in humans and dogs. The 16S amplicon of *Streptococcus mutans* matched that of the human *S. mutans,* with type c identified as the main serotype. This result suggests that human oral bacteria can be transmitted to dogs. Therefore, considering the high frequency of contact between dogs and humans because of communal living and the current poor oral health of dogs, owners must improve the oral hygiene management of their dogs.

## 1. Introduction

In 2022, through an online panel survey on animal protection public awareness among 5000 people nationwide, the Ministry of Agriculture, Food and Rural Affairs of Korea identified that 25.4% of the households were raising companion animals [1]. In addition, the population raising companion animals in Korea was 6.02 million households (29.7% of all households), with 13.06 million people living with an animal. Of these, 80.7% of households had dogs and 25.7% had cats. According to the “2021 Korea Companion Animal Report” published by KB Financial Group’s Management Research Institute, 71.0% of the households raising companion animals had spent money on the treatment of these animals over the past 2 years [2], with the average total medical expenses paid being 232,500 KRW/household/year. These households mostly visited a veterinary hospital for the treatment of skin diseases (44.1%), followed by regular health checkups (34.6%), and the treatment of digestive diseases (24.6%) and dental diseases (23.8%).

According to the US National Institutes of Health, 92% of humans aged 20–64 years experience tooth decay in their permanent teeth [3]. By contrast, the incidence of dental caries was lower in dogs than in humans due to canine tooth morphology, but one or more carious lesions were identified in 5.25% of adult canines [4]. In a caries survey, 23 (5.3%) of 435 dogs developed one or more carious lesions along with significant mineral loss [5]. In dogs, food deposition in the space between the cone-shaped teeth is minor because the space is wide. Additionally, the amylase content of dog saliva is low; therefore, sugar formation through the degradation of deposited food is low. Moreover, the pH of dog saliva is high at 7.5, and its acid-buffering effect is also high; hence, the rate of dental caries is relatively low in dogs [6,7]. A review of large-scale canine dental records revealed the number of dental caries and the location of carious lesions in dogs [5]. Moreover, studies have reported oral bacterial transmission between humans and dogs [8,9]. In fact, 16% of the streptococcal bacteria detected in dogs was positive for *Streptococcus mutans*, and a genetic link was noted between the bacteria found in dogs and their human hosts [10]. On the basis of 16S rRNA sequencing, the G+C content, and DNA–DNA homology analysis of oral samples of 68 domestic dogs, typical *S. mutans* of serotype c was also identified in two dogs [11]. Serotype c *S. mutans* is a serotype highly detected at approximately 70–80% in the human oral cavity. The DNA–DNA hybridization analysis revealed a homology ratio of >90% between the bacteria isolated from the dog and its owner, and the *S. mutans* isolated from the dogs was the same as that from their owners. Thus, these results implied that canine *S. mutans* were transmitted from the owner and that human *S. mutans* could colonize the canine oral cavity.

Instinctively, dogs do not complain of oral disease-induced pain to protect themselves. This lowers the owners’ awareness of the disease, and, subsequently, the oral diseases in their pets are neglected [12]. This factor severely causes chewing disorders and malnutrition and affects the health and lifespan of companion dogs as they can develop bacteremia. Nevertheless, most dog owners are poorly aware of their dogs’ dental caries and periodontal diseases and are careless of oral hygiene practices, such as brushing and scaling [13]. To minimize the risk of human-to-dog bacterial transmission and enhance their dogs’ oral health, it is crucial for owners to be mindful of their dogs’ condition and adhere to regular oral hygiene practices. Furthermore, assessing the owner’s awareness against an expert’s clinical diagnosis proves effective in promoting better oral hygiene behavior. In this study, the genes of dental plaque bacteria isolated from the dogs were also analyzed to support the owners’ needs for improved dog oral hygiene management.

## 2. Materials and Methods

### 2.1. Recruitment of Dogs and Owners

Research cooperation consent was obtained from the Yeah Dental Animal Clinic (Gangnam, Seoul, Korea) for the recruitment of the participating dogs and their owners and for the oral examination of the participating dogs. In all, 19 dog owners (19 dogs) who understood and agreed to the purpose of this study were enrolled in this study. Only dogs who were treated after sedation were recruited in the study. This study was approved by the Institutional Animal Ethics Committee of Eulji University (EUIACUC23-02).

### 2.2. Periodontal Examination of Dogs

The periodontal condition of the dogs was evaluated by measuring their calculi index (CI) and gingiva index (GI), as performed by a veterinarian (Jeong suk Yu) after anesthesia, and the supragingival dental plaque that was removed during scaling was collected for dental plaque bacterial culture. The CI was scored from 0 to 3 at the calculus level based on the modified Ramfjord index [14]. The GI was scored by the level of gingival inflammation from 0 to 3 based on the Löe and Silness gingivitis index [15].

### 2.3. Dog Owner’s Evaluation of the Dog’s Oral Condition

The approach to evaluate the dog’s oral condition from the owner’s perspective included interviewing the owner about the dog’s gender (male, female), licking habits (yes, no), awareness of the oral condition (scores 0, 1, 2, 3), regular checkup (yes, no), scaling experience (yes, no), and tooth brushing frequency (everyday, at least once a week, less than once a month).

### 2.4. Dental Plaque Bacterial Culture and Polymerase Chain Reaction Analysis

Dental plaque around the gingival margin of anesthetized dogs was collected in BHI (Brain Heart Infusion, Becton, Dickinson and Company, Sparks, NV, USA) broth and cultured for 48 hours with shaking in a 5% CO_2_ incubator. The mixed bacterial suspension was incubated until the optical density was 1.2–1.5 (600 nm) and harvested at 5000 rpm. Bacterial genomic DNA extraction was performed using a G-spin genomic DNA extraction kit (for bacteria, iNtRON Biotechnology, Seoung-Nam, Korea) and was then used as a template for PCR analysis. Bacterial genomic DNA (1 μg) plus forward (5’-CGGAGTGCTTTTTACAAGTGCTGG-3’) and reverse primers (5’-AACCACGGCCAGCAAACCCTTTAT-3’) for *S. mutans* serotype c (1 pmole) were mixed with AccuPower^®^ PyroHotStart *Taq* PCR PreMix (Bioneer, Daejeon, Korea). PCR was performed by repeating denaturation (95 °C, 30 s), annealing (60 °C, 30 s), and extension (72 °C, 1 min) 30 times. The PCR reaction solution was electrophoresed on a 1.5% agarose gel, and PCR products were identified on a UV transilluminator. The control *S. mutans* was obtained from the Korean Collection for Type Cultures (Korea Research Institute of Bioscience and Biotechnology, Jeongeup, Korea).

### 2.5. Metagenome Amplicon Sequencing

Metagenome Amplicon Sequencing was performed on 10 out of 19 mixed cultures collected from the dogs’ dental plaque by requesting MACROGEN (Gangnam, Seoul, Korea). DNA was extracted using a DNeasyPowerSoil Kit (Qiagen, Hilden, Germany) according to the manufacturer’s instructions. The extracted DNA was quantified using Quant-IT PicoGreen (Invitrogen, Eugene, OR, USA). The sequencing libraries were prepared according to the Illumina 16S Metagenomic Sequencing Library protocols to amplify the V3 and V4 regions. The input gDNA (2 ng) was PCR amplified with a 5× reaction buffer, 1 mM of dNTP mix, 500 nM each of the universal Forward (F)/Reverse (R) PCR primers, and Herculase II fusion DNA polymerase (Agilent Technologies, Santa Clara, CA). The cycle conditions for the 1st PCR were 3 min at 95 °C for heat activation, and 25 cycles of 30 s at 95 °C, 30 s at 55 °C, and 30 s at 72 °C, followed by a 5 min final extension at 72 °C. The universal primer pair with Illumina adapter overhang sequences used for the first amplifications were as follows: V3-F: 5′-TCGTCGGCAGCGTCAGATGTGTATAAGAGACAGCCTACGGGNGGCWGCAG-3′, V4-R: 5′- GTCTCGTGGGCTCGGAGATGTGTATAAGAGACAGGACTACHVGGGTATCTAATCC-3′. The 1st PCR product was purified with AMPure beads (Agencourt Bioscience, Beverly, MA). Following purification, 2 mL of the 1st PCR product was PCR amplified for a final library construction containing the index using a NexteraXT Indexed Primer. The cycle condition for the 2nd PCR was same as the 1st PCR condition, except for 10 cycles. The PCR product was purified with AMPure beads. The final purified product was then quantified using qPCR according to the qPCR Quantification Protocol Guide (KAPA Library Quantificatoin kits for IlluminaSequecing platforms) and qualified using the TapeStation D1000 ScreenTape (Agilent Technologies, Waldbronn, Germany). The paired-end (2 × 300 bp) sequencing was performed by Macrogen using the MiSeq™ platform (Illumina, San Diego, CA, USA).

### 2.6. Statistical Analysis

Statistical analysis was performed using SPSS 28.0 (SPSS Inc, Chicago, IL, USA). The dog’s gender and licking level were analyzed by frequency and percentage. The CI and GI of dogs are expressed as the mean ± SD. The 19 dogs that participated in the study were categorized into two groups based on whether their teeth were brushed at least once a week by the owners. The relationship between the CI and GI according to the tooth brushing frequency between the two groups was tested using Wilcoxon’s signed-rank test, and the relationship between the owner’s awareness of the dog’s oral condition and tooth brushing frequency was tested by Fisher’s exact test. *p* ≤ 0.05 was considered to indicate statistical significance.

## 3. Results

### 3.1. Owner’s Awareness of Dog’s Oral Condition and the Degree of Tooth Brushing Practice

The owners had to evaluate their awareness level of and the practice related to the dog’s oral condition (Table 1). Among the owners of the 19 participating dogs, 15 owners (78.95%) responded that their dogs frequently licked people’s faces and mouths, whereas 4 dog owners (21.05%) responded that theirs did not (*p* = 0.000). No owner brushed their dog’s teeth every day. Eleven owners brushed their dogs’ teeth more than once a week (57.89%), whereas 8 owners brushed them less than once a month (42.10%). To determine the dog’s oral health on the basis of the tooth brushing frequency, dental plaque on the tooth surface and gingival conditions in the dogs were assessed. A veterinarian determined the dog’s CI and GI. The CI of dogs brushed more than once a week was lower than that of those brushed less than once a month (0.74 ± 0.41 vs. 0.83 ± 0.51). However, the CI exhibited a nonsignificant difference between the two groups (*p* = 0.479 by the Wilcoxon signed-rank test). The GI of dogs brushed more than once a week was lower than that of those brushed less than once a month (0.54 ± 0.36 vs. 0.61 ± 0.47). However, the GI exhibited a nonsignificant difference between the two groups (*p* = 0.840). The perceptions of 12 dog owners about their dogs’ oral condition (63.2%) concurred with the results of a veterinarian’s diagnosis, whereas those of 7 owners were inconsistent (36.8%). Overall, the awareness level of the dog’s oral condition was relatively high (*p* = 0.000). However, we analyzed the relationship between the owner’s brushing frequency and awareness of the dog’s oral condition using Fisher’s exact test and revealed that the brushing frequency was not high, even when the owner was highly aware of the dog’s oral issues (*p* = 0.422).

### 3.2. Genetic Analysis of Dental Plaque Bacteria of Dogs

**Figure 1:**
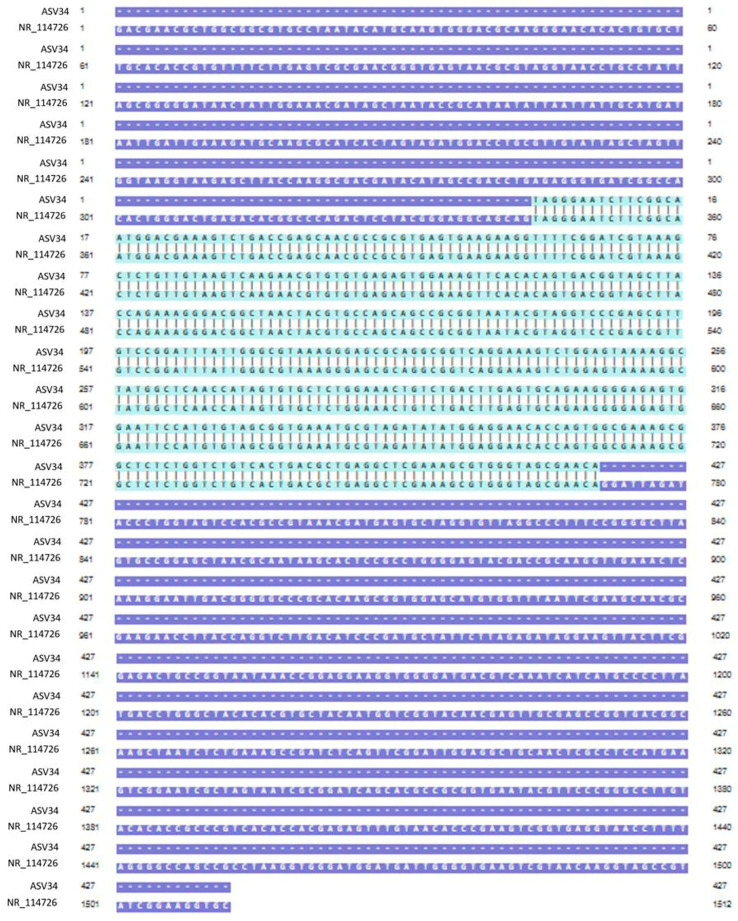
Nucleotide sequence alignment. Alignment of the ASV34 sequence identified from the 16S amplicon of dental plaque bacteria with the oral *S. mutans* sequence reported in GenBank (strain NCTC 10449 16S ribosomal RNA, Accession; NR_114726). The light-green region is the part where the two nucleotide sequences match.

**Figure 2:**
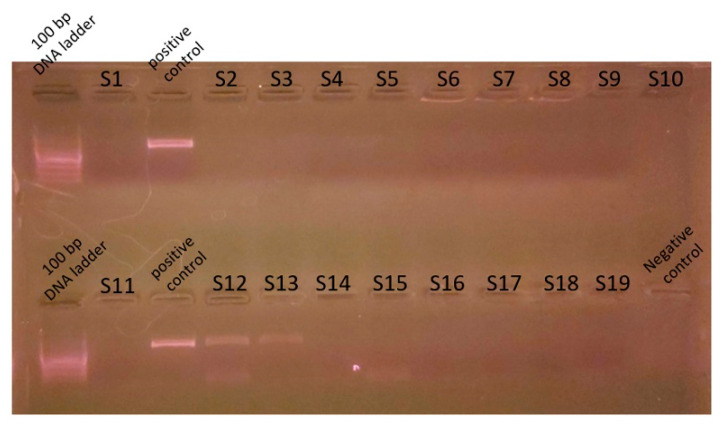
Polymerase chain reaction analysis for *S. mutans*. Genomic DNA was prepared from dental plaque bacteria from dogs and PCR was performed with the *S. mutans* serotype c primer. Nineteen genomic DNA from dental plaque bacteria (S1-S19) were used for the PCR template. Genomic DNA prepared from *S. mutans* strains distributed from the Korean Collection for Type Cultures was used as a control. PCR reaction mixture without a template was used for the negative control. PCR product was analyzed on 1.5% agarose gel electrophoresis. A 100 bp DNA ladder was used as a size marker.

Genomic DNA was extracted from mixed cultures of dental plaque, which were removed during the scaling of 19 dogs. Metagenomic amplicons for 10 of these bacteria were sequenced. Through bioinformatic analysis, 73 species of 16S amplicon sequences were identified from the 10 bacteria (ASV1–ASV76; Supplementary Table S1), except for 3 species whose accession numbers could not be identified (Table 2). Among the 16S amplicon sequences, the sequences of *Staphylococcus intermedius*, *Pateurella canis*, *Pseudomonas aeruginosa*, *Escherichia fergusonii*, and *Pasteurella multocida* were detected at high frequencies. These bacteria are known to cause inflammation, bacteremia, and sepsis in humans. Among the 16S amplicon sequences, the sequence identified as *S. mutans* (ASV34) matched that of the human oral *S. mutans*, as reported in GenBank (strain NCTC 10449 16S ribosomal RNA, Accession: NR_114726) (Figure 1). Genomic DNA obtained from the dental plaque bacteria isolated from the 19 dogs was analyzed through PCR by using primers for serotype c of the human oral *S. mutans*. As shown in Figure 2, the PCR products of the dental plaque bacteria were detected in 2 of the 19 dogs (S12 and S13).

## 4. Discussion

The increase in the number of households raising companion animals has led to an expansion of the market size of various industries, such as those selling companion animal food, health products, and household goods [16]. However, dog owners’ awareness of oral diseases, such as dental caries and periodontal disease, in dogs, is very poor. In general, owners are relatively negligent toward dogs’ oral hygiene [13]. Furthermore, several cases of human-to-dog transmissions of oral disease-risk bacteria have been reported [5,8,9]. We compared the owner’s perception of a dog’s oral health and the degree of oral hygiene practice for dogs with the results of a veterinarian’s clinical diagnosis. We attempted to expose the current problems associated with owners’ dog oral hygiene practices and recommended more improved practices. To rationally support this suggestion, we analyzed the oral disease-related bacteria in dog dental plaque through a genetic analysis.

The chronic disease section of the 2019 Korean National Health Statistics has reported a dental caries rate of 89.1% among adults aged ≥19 years and a dental caries prevalence rate of 33.5% [17]. The dental caries rate is significantly lower in dogs than in humans [4,5,6,7]. However, many studies have reported human-to-dog oral bacterial transmission, including the transmission of *S. mutans*; therefore, the need for improved oral hygiene management in dogs and humans is increasing [8,9]. Considering the high incidence of human dental caries and the risk of human-to-dog oral bacterial transmission, we performed a genetic analysis for identifying canine plaque bacteria. Cavities are formed through demineralization by organic acids produced during the energy metabolism of oral bacteria. In particular, *S. mutans* glycosyltransferase (GTase) binds the glucose moiety of sucrose to α(1-3)- and α(1-6)-links to form the insoluble sugar polymer glucan. Glucan, an extracellular polysaccharide, allows *S. mutans* to attach to the enamel surface and contributes to the additional attachment and stable growth of various bacteria on the enamel surface [18]. Lactic acid secreted around the *S. mutans* attachment site acidifies the environment around the tooth surface, thereby triggering continuous demineralization [18].

Dogs typically recognize and exchange information by licking. Among dogs, licking is the most basic way to seek human attention and express that desire. In total, 85% of dogs lick the hands and 49% lick the faces of their owners [19,20]. Considering the high prevalence of dental caries among human oral diseases and the human-to-dog transmission of *S. mutans*, we studied the presence of *S. mutans* among dental plaque bacteria in dogs [5,8,9,11]. Dental plaque from the participating dogs was first cultured in a media suitable for *S. mutans* growth, followed by 16S amplicon metagenomic sequencing. Therefore, the sequencing results detected only a subset of the dental plaque microbiota and did not ascertain the existence of obligate anaerobic or obligate aerobic bacteria. However, the heightened detection sensitivity of metagenome amplicon sequencing enables the identification of numerous bacterial species, including *S. mutans*. The bioinformatic analysis subsequently revealed 73 species of 16S amplicon sequences. Among these sequences, an amplicon sequence corresponding to *S. mutans* was found to perfectly match that of the human oral *S. mutans* reported in GenBank (Accession: NR_114726). Although we could not directly prove the human-to-dog transmission of *S. mutans* owing to the lack of genetic analyses of oral bacteria from dog owners, these amplicon sequencing results suggested that *S. mutans* in dogs was transmitted from humans. A systematic review and meta-analysis study has proven the mother-to-child vertical transmission of *S. mutans* [21]. Although the exact mechanism underpinning human-to-dog oral bacterial transmission remains unclear, the risk of such a transmission has been suggested in several studies covering a long period [5,8,9,10,11]. Of the streptococcal bacteria detected in dogs, 16% were positive for *S. mutans*. The DNA–DNA hybridization analysis confirmed that the *S. mutans* homology rate between dogs and dog owners was >90%, implying the possibility of human-to-dog transmissions. Based on the chemical composition of serotype-specific rhamnose glucose polymers, *S. mutans* is particularly classified into serotypes c, e, f, and k [22,23,24]. Approximately 70–80% of *S. mutans* detected in the human oral cavity belong to serotype c, with approximately 20% belonging to serotype e and <5% belonging to serotypes f and k [25,26,27]. The presence of *S. mutans* among the dental plaque bacteria of dogs was confirmed through PCR analysis by using a primer for *S. mutans* serotype c. An analysis of Koreans in 2020 revealed that 79.3% of the participants had *S. mutans* serotype c [28]. This serotype is associated with pit and fissure caries and smooth surface caries [29]. Serotype e is detected at a higher level in patients with dental caries, but a higher level of serotype c is detected in plaque on teeth without caries [27]. Dogs lick often. In this cohort, our survey confirmed that 78.95% of the dogs were habituated to lick their owners. The results from our PCR analysis and 16S amplicon metagenomic sequencing, detecting *S. mutans*, provide evidence supporting the possibility of the human-to-dog oral transmission of *S. mutans*.

Through 16S amplicon sequencing, 72 species other than *S. mutans* were detected from the isolated plaque bacteria. According to the bioinformatic analysis, several bacteria capable of causing diseases in humans or dogs were identified from the canine dental plaque. In particular, *P. canis*, *Staphylococcus intermedius*, *Pseudomonas aeruginosa*, *E. fergusonii*, and *Pasteurella multocida* were detected at high rates. *P. canis* causes sepsis, osteomyelitis, and cutaneous abscesses in humans following a dog bite [30,31]. *S. intermedius* is a zoonotic pathogen that can cause skin abscesses in humans after the exposure to animal saliva [32]. Some *Pseudomonas aeruginosa* and *E. fergusonii* strains cause tissue inflammation, bacteremia, and sepsis in immunocompromised humans and are resistant to antibiotics [33,34]. *Pasteurella multocida* causes inflammation (generally diffuse, localized cellulitis) in humans after dog or cat bites. It can also cause bacteremia-induced osteomyelitis or endocarditis [35]. *Treponema denticolar*, a representative human periodontitis risk bacterium, was detected in the subgingival plaque of dogs [9]. Conversely, *Porphyromonas gulae* (previously classified as *Porphyromonas gingivalis*), *Eikenella corrodens*, *Tannerella forsythia*, and *Treponema denticola*, bacteria that cause canine periodontal disease, have been detected in dog owners who were in close contact with their dogs [36]. Human-to-dog transmission of methicillin-resistant *Staphylococcus aureus* has also been reported [37,38]. Despite the difference in the distribution pattern of periodontal disease-causing bacterial species between humans and dogs, several of these species can be transmitted between humans and dogs.

Compared with dental caries bacteria, periodontal disease-causing bacteria are highly active in causing periodontal tissue damage by secreting toxins (such as epitheliotoxin, endotoxin, and leukotoxin) and tissue-degradation enzymes (such as collagenase, gelatinase, and elastase). The excessive production of pro-inflammatory cytokines (interleukin-1, IL-6, IL-8, etc.) from macrophages and fibroblasts in the periodontal tissue because of the ongoing inflammatory response induces the secretion of prostaglandin E2(PGE2) and matrix metalloproteases (MMPs), thereby accelerating periodontal tissue destruction [39]. PGE2 secreted from macrophages around periodontal inflammation tissues particularly induces lymphocytes to secrete the osteoclast activating factor, thereby increasing osteoclast-mediated alveolar bone resorption. Oral microorganisms are primarily commensal bacteria that interact and proliferate in the human mouth and contribute to oral health maintenance by suppressing the growth of harmful bacteria. Therefore, appropriate oral health care is crucial for maintaining normal bacterial flora [40]. However, depending on the oral hygiene status and health, harmful bacteria can proliferate, commensal bacteria can become pathogenic, and the external infection risk can increase. Therefore, oral hygiene is aimed at bacterial management for maintaining healthy oral flora.

Because the owner is responsible for managing the dog’s oral hygiene, an expert must clinically determine whether the owner’s perception of the dog’s oral health status is correct. The CI and GI are basic clinical tools used for diagnosing dental caries and periodontitis. Contrary to the veterinarian’s diagnosis, if the owner’s awareness about the dog’s oral health status is inaccurate, the dog’s oral hygiene will be neglected. However, a significant number of participating owners (63.2%) were aware of their dogs’ oral condition, and their awareness was consistent with the results of the veterinarian’s diagnosis. Nevertheless, oral hygiene practices followed by owners for their dogs were very poor. Furthermore, owners were aware of several oral health problems in dogs. However, no participating dog owner performed daily brushing, albeit a proportion did so several times weekly or monthly. These findings prove that, despite the high recognition of the oral condition-related problems of dogs, the behavior of dog owners in maintaining the oral hygiene of dogs remains poor. Additionally, the average CI and GI values were lower in dogs brushed more than once a week than in dogs brushed less than once a month, albeit the difference between these CI and GI values was nonsignificant. This finding implies that the owner brushes the dog’s teeth less frequently, which makes brushing ineffective. A study on 50 dogs reported results consistent with our study [13]. The high awareness of oral hygiene-related problems in owners did not successfully translate into regular tooth brushing practices of their pets [13]. Most dog owners brushed their dogs’ teeth <5 times in 3 months and did not perform scaling. Thus, the oral hygiene practice was very poor. Animals do not characteristically complain of pain because of their protective instincts. Consequently, most dog owners generally recognize the dogs’ oral health problems late or neglect them [12]. Therefore, the inadequate and inaccurate recognition of the dog’s oral problems by the dog owner can result in poor dog oral hygiene behavior. In addition, mesophilic aerobic bacteria and enteric bacteria have been detected in dog food and food bowls, so it is necessary to minimize cross-infection and microbial contamination by humans to manage dog oral hygiene [41]. Addressing issues in dog hygiene management requires enhancing owners’ awareness through proactive education on dog oral hygiene by veterinarians and disseminating information widely through various media channels [42].

## 5. Conclusions

We observed that dog owners’ oral hygiene behavior was very poor despite them being highly aware of their dogs’ oral health status. Additionally, various oral disease-related bacteria in dogs and humans were identified in dog dental plaque through a genetic analysis. The amplicon sequence of *S. mutans* detected in the dogs’ dental plaque was identical to the nucleotide sequence of *S. mutans* from the human oral cavity. Further studies involving a large sample size of dogs and genetic analysis of the owners’ oral bacteria are warranted to complement our amplicon sequencing results that offer complementary data for the possibility of human-to-dog oral bacterial transmission. For a dog’s oral health, the owners must urgently recognize their dogs’ oral health status appropriately and practice significantly improved oral hygiene practices.

## Figures and Tables

**Table 1:** Clinical diagnosis of dogs’ oral health by veterinarians and oral hygiene practices and awareness of dogs’ owners.

Category	Response	Unit		*p* Value
Gender	Male	9 (47.36%)		>0.05 *
	Female	10 (52.64%)		
Tendency to lick people frequently	Yes	n = 15 (78.95%)		0.000 *
	No	n = 4 (21.05%)		
Oral hygiene of dogs	Tooth brushing at least once a week (N = 11, 57.89%)	CI	0.74 ± 0.41 ^〒^	CI; 0.479 * GI; 0.840 *
		GI	0.54 ± 0.36 ^〒^	
	Tooth brushing less than once a month (N = 8, 42.10%)	CI	0.83 ± 0.51 ^〒^	
		GI	0.61 ± 0.47 ^〒^	
Correlation between dog’s clinical diagnosis vs. the owner’s awareness of the oral condition		yes (n = 12, 63.2%)		0.000 *
		no (n = 7, 36.8%)		
Tooth brushing frequency vs. the dog owner’s awareness of the dog’s oral condition				0.422 ^#^

^〒^ Mean ± SD. * *p*-value was determined from Wilcoxon signed-rank test. **^#^** *p*-value was determined from Fisher’s exact test.

**Table 2:** List of bacterial species identified through metagenome amplicon sequencing. The 16S amplicon of bacteria obtained from mixed cultures of dental plaque was analyzed for bacterium identification (n = 10).

Group	Kingdom	Phylum	Class	Order	Family	Genus	Species	Accession No.	SI	S2	S4	S5	S8	S9	S12	S13	S15	S18
ASV1	*Bacteria*	*Bacillota*	*Bacilli*	*Bacillales*	*Staphylococcaceae*	*Staphylococcus*	*Staphylococcus intermedius*	NR_036829.1	23,919	53,961	0	40,926	38,350	0	27,799	55,779	27,311	46
ASV2	*Bacteria*	*Proteobacteria*	*Gammaproteobacteria*	*Pseudomonadales*	*Pseudomonadaceae*	*Pseudomonas*	*Pseudomonas aeruginosa*	NR_113599.1	0	0	0	0	0	0	0	0	0	54,196
ASV3	*Bacteria*	*Actinomycetota*	*Actinomycetes*	*Micrococcales*	*Micrococcaceae*	*Micrococcus*	*Micrococcus aloeverae*	NR_134088.1	0	0	0	0	0	54,304	0	0	0	0
ASV4	*Bacteria*	*Proteobacteria*	*Gammaproteobacteria*	*Pasteurellales*	*Pasteurellaceae*	*Pasteurella*	*Pasteurella canis*	NR_042882.1	658	603	11,342	13,574	15,373	0	0	0	0	0
ASV5	*Bacteria*	*Bacillota*	*Bacilli*	*Bacillales*	*Bacillaceae*	*Bacillus*	*Bacillus clarus*	NR_180213.1	0	0	35,677	0	0	0	0	0	0	0
ASV6	*Bacteria*	*Proteobacteria*	*Gammaproteobacteria*	*Enterobacterales*	*Enterobacteriaceae*	*Escherichia*	*Escherichia fergusonii*	NR_114079.1	29,916	0	0	0	0	6	0	0	0	2
ASV7	*Bacteria*	*Proteobacteria*	*Gammaproteobacteria*	*Moraxellales*	*Moraxellaceae*	*Acinetobacter*	*Acinetobacter geminorum*	NR_181169.1	0	0	0	0	0	0	0	0	21,760	0
ASV8	*Bacteria*	*Proteobacteria*	*Gammaproteobacteria*	*Pasteurellales*	*Pasteurellaceae*	*Pasteurella*	*Pasteurella multocida*	NR_115138.1	0	0	0	0	0	0	12,118	0	0	0
ASV9	*Bacteria*	*Proteobacteria*	*Gammaproteobacteria*	*Pasteurellales*	*Pasteurellaceae*	*Pasteurella*	*Pasteurella multocida*	NR_115138.1	0	0	0	0	0	0	1632	0	0	0
ASV10	*Bacteria*	*Proteobacteria*	*Gammaproteobacteria*	*Pasteurellales*	*Pasteurellaceae*	*Pasteurella*	*Pasteurella stomatis*	NR_042888.1	0	0	6178	0	0	0	0	0	0	0
ASV11	*Bacteria*	*Bacillota*	*Bacilli*	*Bacillales*	*Staphylococcaceae*	*Staphylococcus*	*Staphylococcus intermedius*	NR_036829.1	0	0	0	0	0	0	0	0	5558	0
ASV12	*Bacteria*	*Bacillota*	*Bacilli*	*Bacillales*	*Staphylococcaceae*	*Staphylococcus*	*Staphylococcus intermedius*	NR_036829.1	541	1314	0	1006	966	0	766	0	718	0
ASV13	*Bacteria*	*Proteobacteria*	*Gammaproteobacteria*	*Pasteurellales*	*Pasteurellaceae*	*Pasteurella*	*Pasteurella multocida*	NR_115138.1	0	0	0	0	0	0	2171	0	0	0
ASV14	*Bacteria*	*Proteobacteria*	*Gammaproteobacteria*	*Pasteurellales*	*Pasteurellaceae*	*Pasteurella*	*Pasteurella multocida*	NR_115137.1	0	0	0	0	0	1	2076	0	0	5
ASV15	*Bacteria*	*Proteobacteria*	*Gammaproteobacteria*	*Pseudomonadales*	*Pseudomonadaceae*	*Pseudomonas*	*Pseudomonas aeruginosa*	NR_113599.1	0	0	0	0	0	0	0	0	0	1275
ASV16	*Bacteria*	*Actinomycetota*	*Actinomycetes*	*Micrococcales*	*Micrococcaceae*	*Micrococcus*	*Micrococcus aloeverae*	NR_134088.1	0	0	0	0	0	1414	0	0	0	0
ASV17	*Bacteria*	*Proteobacteria*	*Gammaproteobacteria*	*Pasteurellales*	*Pasteurellaceae*	*Pasteurella*	*Pasteurella stomatis*	NR_042888.1	0	0	1105	0	0	0	0	0	0	0
ASV18	*Bacteria*	*Proteobacteria*	*Gammaproteobacteria*	*Pasteurellales*	*Pasteurellaceae*	*Pasteurella*	*Pasteurella canis*	NR_042882.1	0	0	0	370	401	0	0	0	0	0
ASV19	*Bacteria*	*Proteobacteria*	*Gammaproteobacteria*	*Enterobacterales*	*Enterobacteriaceae*	*Escherichia*	*Escherichia fergusonii*	NR_114079.1	780	0	0	0	0	0	0	0	0	0
ASV20	*Bacteria*	*Proteobacteria*	*Gammaproteobacteria*	*_Moraxellales*	*Moraxellaceae*	*Acinetobacter*	*Acinetobacter geminorum*	NR_181169.1	0	0	0	0	0	0	0	0	533	0
ASV21	*Bacteria*	*Proteobacteria*	*Gammaproteobacteria*	*Enterobacterales*	*Erwiniaceae*	*Kalamiella*	*Kalamiella piersonii*	NR_181783.1	0	0	454	0	0	0	0	0	0	0
ASV22	*Bacteria*	*Bacillota*	*Bacilli*	*Bacillales*	*Bacillaceae*	*Bacillus*	*Bacillus clarus*	NR_180213.1	0	0	346	0	0	0	0	0	0	0
ASV23	*Bacteria*	*Bacillota*	*Bacilli*	*Bacillales*	*Staphylococcaceae*	*Staphylococcus*	*Staphylococcus cohnii*	NR_036902.1	0	0	298	0	0	0	0	0	0	0
ASV24	*Bacteria*	*Proteobacteria*	*Gammaproteobacteria*	*Pasteurellales*	*Pasteurellaceae*	*Pasteurella*	*Pasteurella multocida*	NR_115138.1	0	0	0	0	0	0	308	0	0	0
ASV25	*Bacteria*	*Bacillota*	*Bacilli*	*Bacillales*	*Staphylococcaceae*	*Staphylococcus*	*Staphylococcus simulans*	NR_036906.1	0	0	0	0	266	0	0	0	0	0
ASV26	*Bacteria*	*Bacillota*	*Bacilli*	*Bacillales*	*Staphylococcaceae*	*Staphylococcus*	*Staphylococcus agnetis*	NR_117863.1	0	0	0	0	255	0	0	0	0	0
ASV27	*Bacteria*	*Bacillota*	*Bacilli*	*Bacillales*	*Bacillaceae*	*Bacillus*	*Bacillus clarus*	NR_180213.1	0	0	187	0	0	0	0	0	0	0
ASV28	*Bacteria*	*Bacillota*	*Bacilli*	*Lactobacillales*	*Enterococcaceae*	*Enterococcus*	*Enterococcus hirae*	NR_114783.2	0	0	0	0	242	0	0	0	0	0
ASV29	*Bacteria*	*Proteobacteria*	*Gammaproteobacteria*	*Pseudomonadales*	*Pseudomonadaceae*	*Pseudomonas*	*Pseudomonas aeruginosa*	NR_113599.1	0	0	0	0	0	0	0	0	0	145
ASV30	*Bacteria*	*Bacillota*	*Bacilli*	*Bacillales*	*Staphylococcaceae*	*Staphylococcus*	*Staphylococcus ureilyticus*	NR_037046.1	0	0	117	0	0	0	0	0	0	0
ASV31	*Bacteria*	*Proteobacteria*	*Gammaproteobacteria*	*Pseudomonadales*	*Pseudomonadaceae*	*Pseudomonas*	*Pseudomonas aeruginosa*	NR_113599.1	0	0	0	0	0	0	0	0	0	94
ASV32	*Bacteria*	*Proteobacteria*	*Gammaproteobacteria*	*Enterobacterales*	*Erwiniaceae*	*Kalamiella*	*Kalamiella piersonii*	NR_181783.1	0	0	99	0	0	0	0	0	0	0
ASV33	*Bacteria*	*Bacillota*	*Bacilli*	*Bacillales*	*Staphylococcaceae*	*Staphylococcus*	*Staphylococcus canis*	NR_181183.1	0	0	0	0	0	0	0	63	0	0
ASV34	*Bacteria*	*Bacillota*	*Bacilli*	*Lactobacillales*	*Streptococcaceae*	*Streptococcus*	*Streptococcus mutans*	NR_114726.1	0	0	0	0	0	0	8	3	0	0
ASV35	*Bacteria*	*Proteobacteria*	*Alphaproteobacteria*	*Hyphomicrobiales*	*Phyllobacteriaceae*	*Phyllobacterium*	*Phyllobacterium zundukense*	NR_181634.1	0	2	0	0	0	39	0	0	0	30
ASV36	*Bacteria*	*Actinomycetota*	*Actinomycetes*	*Micrococcales*	*Micrococcaceae*	*Micrococcus*	*Micrococcus aloeverae*	NR_134088.1	0	0	0	0	0	68	0	0	0	0
ASV37	*Bacteria*	*Bacillota*	*Bacilli*	*Bacillales*	*Staphylococcaceae*	*Staphylococcus*	*Staphylococcus cohnii*	NR_036902.1	0	0	59	0	0	0	0	0	0	0
ASV38	*Bacteria*	*Proteobacteria*	*Gammaproteobacteria*	*Enterobacterales*	*Enterobacteriaceae*	*Escherichia*	*Escherichia fergusonii*	NR_114079.1	66	0	0	0	0	0	0	0	0	0
ASV39	*Bacteria*	*Bacteroidota*	*Chitinophagia*	*Chitinophagales*	*Chitinophagaceae*	*Hydrotalea*	*Hydrotalea flava*	NR_117026.1	0	0	0	0	0	25	0	0	0	17
ASV40	*Bacteria*	*Proteobacteria*	*Alphaproteobacteria*	*Hyphomicrobiales*	*Phyllobacteriaceae*	*Mesorhizobium*	*Mesorhizobium terrae*	NR_180479.1	0	0	0	0	0	7	0	0	0	7
ASV41	*Bacteria*	*Bacillota*	*Bacilli*	*Bacillales*	*Staphylococcaceae*	*Staphylococcus*	*Staphylococcus lugdunensis*	NR_024668.1	0	0	15	0	0	0	0	0	0	0
ASV42	*Bacteria*	*Proteobacteria*	*Betaproteobacteria*	*Neisseriales*	*Neisseriaceae*	*Neisseria*	*Neisseria zoodegmatis*	NR_043459.1	0	0	0	0	0	0	0	10	0	0
ASV43	*Bacteria*	*Bacillota*	*Bacilli*	*Lactobacillales*	*Enterococcaceae*	*Enterococcus*	*Enterococcus lactis*	NR_117562.1	0	0	0	0	16	0	0	0	0	0
ASV44	*Bacteria*	*Proteobacteria*	*Betaproteobacteria*	*Neisseriales*	*Neisseriaceae*	*Neisseria*	*Neisseria animaloris*	NR_043458.1	0	0	0	0	0	0	0	5	0	0
ASV45	*Bacteria*	*-*	*-*	*-*	*-*	*-*	*-*	-	0	0	0	0	0	0	0	0	0	9
ASV46	*Bacteria*	*Cyanobacteria*	*-*	*Oscillatoriales*	*-*	*Potamosiphon*	*Potamosiphon australiensis*	NR_177904.1	0	0	0	0	0	0	0	0	0	6
ASV47	*Bacteria*	*Actinomycetota*	*Actinomycetes*	*Micrococcales*	*Micrococcaceae*	*Micrococcus*	*Micrococcus luteus*	NR_075062.2	0	0	0	0	0	0	0	5	0	0
ASV48	*Bacteria*	*Cyanobacteria*	*-*	*Oscillatoriales*	*-*	*Potamosiphon*	*Potamosiphon australiensis*	NR_177904.1	0	0	0	0	0	10	0	0	0	0
ASV49	*Bacteria*	*Bacillota*	*Bacilli*	*Bacillales*	*Bacillaceae*	*Calidifontibacillus*	*Calidifontibacillus erzurumensis*	NR_180225.1	0	0	0	0	0	0	0	0	0	5
ASV50	*Bacteria*	*Proteobacteria*	*Gammaproteobacteria*	*Pseudomonadales*	*Pseudomonadaceae*	*Pseudomonas*	*Pseudomonas aeruginosa*	NR_113599.1	0	0	0	0	0	0	0	0	0	5
ASV51	*Bacteria*	*Bacillota*	*Bacilli*	*Bacillales*	*Staphylococcaceae*	*Staphylococcus*	*Staphylococcus muscae*	NR_104762.1	0	0	0	0	0	0	0	3	0	0
ASV52	*Bacteria*	*Proteobacteria*	*Epsilonproteobacteria*	*Campylobacterales*	*Campylobacteraceae*	*Campylobacter*	*Campylobacter massiliensis*	NR_181373.1	0	0	0	0	7	0	0	0	0	0
ASV53	*Bacteria*	*Proteobacteria*	*Alphaproteobacteria*	*Hyphomicrobiales*	*Nitrobacteraceae*	*Bradyrhizobium*	*Bradyrhizobium australafricanum*	NR_180493.1	0	0	0	0	0	0	0	0	0	3
ASV54	*Bacteria*	*Proteobacteria*	*Alphaproteobacteria*	*Hyphomicrobiales*	*Xanthobacteraceae*	*Labrys*	*Labrys wisconsinensis*	NR_116004.1	0	0	0	0	0	0	0	0	0	5
ASV55	*Bacteria*	*Bacteroidota*	*Chitinophagia*	*Chitinophagales*	*Chitinophagaceae*	*Chitinophaga*	*Chitinophaga vietnamensis*	NR_180543.1	0	0	0	0	0	3	0	0	0	0
ASV56	*Bacteria*	*Proteobacteria*	*Betaproteobacteria*	*Burkholderiales*	*Comamonadaceae*	*Variovorax*	*Variovorax boronicumulans*	NR_114214.1	0	0	0	0	0	2	0	0	0	1
ASV57	*Bacteria*	*Bacillota*	*Bacilli*	*Bacillales*	*Staphylococcaceae*	*Staphylococcus*	*Staphylococcus intermedius*	NR_036829.1	0	0	0	0	0	0	0	0	0	3
ASV58	*Bacteria*	*Proteobacteria*	*Gammaproteobacteria*	*Enterobacterales*	*Erwiniaceae*	*Pantoea*	*[Curtobacterium] plantarum*	NR_104943.1	0	0	0	0	0	0	0	0	0	3
ASV59	*Bacteria*	*Proteobacteria*	*Gammaproteobacteria*	*Pseudomonadales*	*Pseudomonadaceae*	*Pseudomonas*	*Pseudomonas nicosulfuronedens*	NR_180597.1	0	0	0	0	0	0	0	0	0	3
ASV60	*Bacteria*	*Bacillota*	*Bacilli*	*Bacillales*	*Staphylococcaceae*	*Staphylococcus*	*Staphylococcus agnetis*	NR_117863.1	0	0	0	0	0	0	0	0	0	5
ASV61	*Bacteria*	*Proteobacteria*	*Gammaproteobacteria*	*Pseudomonadales*	*Pseudomonadaceae*	*Pseudomonas*	*Pseudomonas aeruginosa*	NR_113599.1	0	0	0	0	0	0	0	0	0	4
ASV62	*Bacteria*	*-*	*-*	*-*	*-*	*-*	*-*	-	0	0	0	0	0	0	0	0	0	5
ASV63	*Bacteria*	*Bacillota*	*Bacilli*	*Bacillales*	*Staphylococcaceae*	*Staphylococcus*	*Staphylococcus durrellii*	NR_181502.1	0	0	0	0	0	0	0	3	0	0
ASV64	*Bacteria*	*Bacillota*	*Bacilli*	*Bacillales*	*Staphylococcaceae*	*Staphylococcus*	*Staphylococcus chromogenes*	NR_036901.1	0	0	0	0	0	0	0	2	0	0
ASV65	*Bacteria*	*Bacillota*	*Bacilli*	*Bacillales*	*Bacillaceae*	*Halalkalibacterium*	*Halalkalibacterium halodurans*	NR_025446.1	0	0	0	4	0	0	0	0	0	0
ASV66	*Bacteria*	*Bacillota*	*Bacilli*	*Bacillales*	*Staphylococcaceae*	*Staphylococcus*	*Staphylococcus simulans*	NR_036906.1	0	0	0	0	4	0	0	0	0	0
ASV67	*Bacteria*	*Bacillota*	*Bacilli*	*Bacillales*	*Staphylococcaceae*	*Staphylococcus*	*Staphylococcus lutrae*	NR_036791.1	0	0	0	0	0	0	0	2	0	0
ASV68	*Bacteria*	*Proteobacteria*	*Gammaproteobacteria*	*Pasteurellales*	*Pasteurellaceae*	*Pasteurella*	*Pasteurella canis*	NR_042882.1	0	0	0	0	0	0	0	0	0	2
ASV69	*Bacteria*	*Proteobacteria*	*Gammaproteobacteria*	*Pseudomonadales*	*Pseudomonadaceae*	*Pseudomonas*	*Pseudomonas glycinis*	NR_181729.1	0	0	0	0	0	0	0	1	0	0
ASV70	*Bacteria*	*-*	*-*	*-*	*-*	*-*	*-*	-	0	0	0	0	0	0	0	0	0	0
ASV71	*Bacteria*	*Proteobacteria*	*Alphaproteobacteria*	*Caulobacterales*	*Caulobacteraceae*	*Brevundimonas*	*Brevundimonas poindexterae*	NR_114709.1	0	0	0	0	0	1	0	0	0	1
ASV72	*Bacteria*	*Actinomycetota*	*Actinomycetes*	*Corynebacteriales*	*Mycobacteriaceae*	*Mycobacterium*	*Mycobacterium numidiamassiliense*	NR_179524.1	0	0	0	0	0	0	0	0	0	1
ASV73	*Bacteria*	*Bacillota*	*Bacilli*	*Lactobacillales*	*Lactobacillaceae*	*Lacticaseibacillus*	*Lacticaseibacillus baoqingensis*	NR_180279.1	0	0	0	0	0	0	0	0	0	2
ASV74	*Bacteria*	*Bacillota*	*Bacilli*	*Bacillales*	*Bacillaceae*	*Bacillus*	*Bacillus rhizoplanae*	NR_181926.1	0	0	1	0	0	0	0	0	0	0
ASV75	*Bacteria*	*Bacillota*	*Bacilli*	*Bacillales*	*Bacillaceae*	*Bacillus*	*Bacillus clarus*	NR_180213.1	0	0	2	0	0	0	0	0	0	0
ASV76	*Bacteria*	*Proteobacteria*	*Proteobacteria*	*Pseudomonadales*	*Pseudomonadaceae*	*Pseudomonas*	*Pseudomonas migulae*	NR_114223.1	0	0	0	0	0	0	0	2	0	0

## Data Availability

The data and materials of this article are included within the article. The data supporting the findings of this study are available from the corresponding author upon reasonable request.

## Supplementary Materials

The following supporting information can be downloaded at: https://www.mdpi.com/article/10.3390/vetsci11020096/s1, Table S1: 16S amplicon sequences for 73 species bacteria.
