# Supplementary material for: Evidence Supporting Oral Hygiene Management by Owners through a Genetic Analysis of Dental Plaque Bacteria in Dogs
Source: Vet Sci. 2024 Feb 19;11(2):96. doi: 10.3390/vetsci11020096 (PMC10893504; doi:10.3390/vetsci11020096)
Supplement: Supplementary file 1 [file vetsci-11-00096-s001.zip › vetsci-2851398-supplementary.pdf]

Table S1. 16S amplicon sequences for 73 species bacteria.

>ASV1
TAGGGAATCTTCCGCAATGGGCGAAAGCCTGACGGAGCAACGCCGCGTGAGTGATGAAGGTCTTCGGATCGTAAAGCTCTGT
TGTTAGGGAAGAACAAATGTGTAAGTAACTGTGCACATCTTGACGGTACCTAACCAGAAAGCCACGGCTAACTACGTGCCAGC
AGCCGCGGTAATACGTAGGTGGCAAGCGTTATCCGGAATTATTGGGCGTAAAGCGCGCGTAGGCGGTTTTTTAAGTCTGATGT
GAAAGCCCACGGCTCAACCGTGGAGGGTCATTGGAAACTGGAAAACTTGAGTGCAGAAGAGGAAAGTGGAATTCCATGTGTA
GCGGTGAAATGCGCAGAGATATGGAGGAACACCAGTGGCGAAGGCGGCTTTCTGGTCTGCAACTGACGCTGATGTGCGAAAG
CGTGGGGATCAAACA

>ASV2
TGGGGAATATTGGACAATGGGCGAAAGCCTGATCCAGCCATGCCGCGTGTGTGAAGAAGGTCTTCGGATTGTAAAGCACTTTA
AGTTGGGAGGAAGGGCAGTAAGTTAATACCTTGCTGTTTTGACGTTACCAACAGAATAAGCACCGGCTAACTTCGTGCCAGCA
GCCGCGGTAATACGAAGGGTGCAAGCGTTAATCGGAATTACTGGGCGTAAAGCGCGCGTAGGTGGTTCAGCAAGTTGGATGT
GAAATCCCCGGGCTCAACCTGGGAACTGCATCCAAAACTACTGAGCTAGAGTACGGTAGAGGGTGGTGGAATTTCCTGTGTAG
CGGTGAAATGCGTAGATATAGGAAGGAACACCAGTGGCGAAGGCGACCACCTGGACTGATACTGACACTGAGGTGCGAAAGC
GTGGGGAGCAAACA

>ASV3
TGGGGAATATTGCACAATGGGCGCAAGCCTGATGCAGCGACGCCGCGTGAGGGATGACGGCCTTCGGGTTGTAAACCTCTTT
CAGTAGGGAAGAAGCGAAAGTGACGGTACCTGCAGAAGAAGCACCGGCTAACTACGTGCCAGCAGCCGCGGTAATACGTAGG
GTGCGAGCGTTATCCGGAATTATTGGGCGTAAAGAGCTCGTAGGCGGTTTGTCGCGTCTGTCGTGAAAGTCCGGGGCTTAACC
CCGGATCTGCGGTGGGTACGGGCAGACTAGAGTGCAGTAGGGGAGACTGGAATTCCTGGTGTAGCGGTGGAATGCGCAGATA
TCAGGAGGAACACCGATGGCGAAGGCAGGTCTCTGGGCTGTAACTGACGCTGAGGAGCGAAAGCATGGGGAGCGAACA

>ASV4
TGGGGAATATTGCGCAATGGGGGGAACCCTGACGCAGCCATGCCGCGTGAATGAAGAAGGCCTTCGGGTTGTAAAGTTCTTT
CGGTGATGAGGAAGGGGTATTATTGAATAGATAATATCATTGACGTTAATTACAGAAGAAGCACCGGCTAACTCCGTGCCAGC
AGCCGCGGTAATACGGAGGGTGCGAGCGTTAATCGGAATAACTGGGCGTAAAGGGCACGCAGGCGGACTTTTAAGTGAGATG
TGAAATCCCCGAGCTTAACTTGGGAACTGCATTTCAGACTGGGAGTCTAGAGTACTTTAGGGAGGGGTAGAATTCCACGTGTA
GCGGTGAAATGCGTAGAGATGTGGAGGAATACCGAAGGCGAAGGCAGCCCCTTGGGAATGTACTGACGCTCATGTGCGAAAG
CGTGGGGAGCAAACA

>ASV5
TAGGGAATCTTCCGCAATGGACGAAAGTCTGACGGAGCAACGCCGCGTGAGTGATGAAGGCTTTCGGGTCGTAAAACTCTGTT
GTTAGGGAAGAACAAGTGCTAGTTGAATAAGCTGGCACCTTGACGGTACCTAACCAGAAAGCCACGGCTAACTACGTGCCAG
CAGCCGCGGTAATACGTAGGTGGCAAGCGTTATCCGGAATTATTGGGCGTAAAGCGCGCGCAGGTGGTTTCTTAAGTCTGATG
TGAAAGCCCACGGCTCAACCGTGGAGGGTCATTGGAAACTGGGAGACTTGAGTGCAGAAGAGGAAAGTGGAATTCCATGTGT
AGCGGTGAAATGCGTAGAGATATGGAGGAACACCAGTGGCGAAGGCGACTTTCTGGTCTGTAACTGACACTGAGGCGCGAAA
GCGTGGGGAGCAAACA

>ASV6
TGGGGAATATTGCACAATGGGCGCAAGCCTGATGCAGCCATGCCGCGTGTATGAAGAAGGCCTTCGGGTTGTAAAGTACTTTC
AGCGGGGAGGAAGGGAGTAAAGTTAATACCTTTGCTCATTGACGTTACCCGCAGAAGAAGCACCGGCTAACTCCGTGCCAGC
AGCCGCGGTAATACGGAGGGTGCAAGCGTTAATCGGAATTACTGGGCGTAAAGCGCACGCAGGCGGTTTGTTAAGTCAGATG
TGAAATCCCCGGGCTCAACCTGGGAACTGCATCTGATACTGGCAAGCTTGAGTCTCGTAGAGGGGGGTAGAATTCCAGGTGTA
GCGGTGAAATGCGTAGAGATCTGGAGGAATACCGGTGGCGAAGGCGGCCCCCTGGACGAAGACTGACGCTCAGGTGCGAAAG
CGTGGGGAGCAAACA

>ASV7
TGGGGAATATTGGACAATGGGCGCAAGCCTGATCCAGCCATGCCGCGTGTGTGAAGAAGGCCTTATGGTTGTAAAGCACTTTA
AGCGAGGAGGAGGCTACTTTAGATAATACCTAGAGATAGTGGACGTTACTCGCAGAATAAGCACCGGCTAACTCTGTGCCAGC
AGCCGCGGTAATACAGAGGGTGCAAGCGTTAATCGGATTTACTGGGCGTAAAGCGCGCGTAGGCGGCTAATTAAGTCAAATG
TGAAATCCCCGAGCTTAACTTGGGAATTGCATTCGATACTGGTTAGCTAGAGTGTGGGAGAGGATGGTAGAATTCCAGGTGTA
GCGGTGAAATGCGTAGAGATCTGGAGGAATACCGATGGCGAAGGCAGCCATCTGGCCTAACACTGACGCTGAGGTGCGAAAG
CATGGGGAGCAAACA

>ASV8
TGGGGAATATTGCGCAATGGGGGGAACCCTGACGCAGCCATGCCGCGTGAATGAAGAAGGCCTTCGGGTTGTAAAGTTCTTT
CGGTAATGAGGAAGGGATGTTGTTTAATAGATAGCATCATTGACGTTAATTACAGAAGAAGCACCGGCTAACTCCGTGCCAGC
AGCCGCGGTAATACGGAGGGTGCGAGCGTTAATCGGAATAACTGGGCGTAAAGGGCACGCAGGCGGACTTTTAAGTGAGATG
TGAAATCCCCGAGCTTAACTTGGGAATTGCATTTCAGACTGGGAGTCTAGAGTACTTTAGGGAGGGGTAGAATTCCACGTGTA
GCGGTGAAATGCGTAGAGATGTGGAGGAATACCGAAGGCGAAGGCAGCCCCTTGGGAATGTACTGACGCTCATGTGCGAAAG
CGTGGGGAGCAAACA

>ASV9
TGGGGAATATTGCGCAATGGGGGGAACCCTGACGCAGCCATGCCGCGTGAATGAAGAAGGCCTTCGGGTTGTAAAGTTCTTT
CGGTAATGAGGAAGGGATGTTGTTTAATAGATGGCATCATTGACGTTAATTACAGAAGAAGCACCGGCTAACTCCGTGCCAGC
AGCCGCGGTAATACGGAGGGTGCGAGCGTTAATCGGAATAACTGGGCGTAAAGGGCACGCAGGCGGACTTTTAAGTGAGATG
TGAAATCCCCGAGCTTAACTTGGGAATTGCATTTCAGACTGGGAGTCTAGAGTACTTTAGGGAGGGGTAGAATTCCACGTGTA
GCGGTGAAATGCGTAGAGATGTGGAGGAATACCGAAGGCGAAGGCAGCCCCTTGGGAATGTACTGACGCTCATGTGCGAAAG
CGTGGGGAGCAAACA

>ASV10
TGGGGAATATTGCGCAATGGGGGGAACCCTGACGCAGCCATGCCGCGTGAATGAAGAAGGCCTTCGGGTTGTAAAGTTCTTT
CGGTAATGAGGAAGGGGTATTATTGAATAGATAATATCATTGACGTTAATTACAGAAGAAGCACCGGCTAACTCCGTGCCAGC
AGCCGCGGTAATACGGAGGGTGCGAGCGTTAATCGGAATAACTGGGCGTAAAGGGCACGCAGGCGGACTTTTAAGTGAGATG
TGAAATCCCCGAGCTTAACTTGGGAACTGCATTTCAGACTGGGAGTCTAGAGTACTTTAGGGAGGGGTAGAATTCCACGTGTA
GCGGTGAAATGCGTAGAGATGTGGAGGAATACCGAAGGCGAAGGCAGCCCCTTGGGAATGTACTGACGCTCATGTGCGAAAG
CGTGGGGAGCAAACA

>ASV11
TAGGGAATCTTCCGCAATGGGCGAAAGCCTGACGGAGCAACGCCGCGTGAGTGATGAAGGTCTTCGGATCGTAAAGCTCTGT
TGTTAGGGAAGAACAAATGTGTAAGTAACTGTGTACATCTTGACGGTACCTAACCAGAAAGCCACGGCTAACTACGTGCCAGC
AGCCGCGGTAATACGTAGGTGGCAAGCGTTATCCGGAATTATTGGGCGTAAAGCGCGCGTAGGCGGTTTTTTAAGTCTGATGT
GAAAGCCCACGGCTCAACCGTGGAGGGTCATTGGAAACTGGAAAACTTGAGTGCAGAAGAGGAAAGTGGAATTCCATGTGTA
GCGGTGAAATGCGCAGAGATATGGAGGAACACCAGTGGCGAAGGCGGCTTTCTGGTCTGCAACTGACGCTGATGTGCGAAAG
CGTGGGGATCAAACA

>ASV12
TAGGGAATCTTCCGCAATGGGCGAAAGCCTGACGGAGCAACGCCGCGTGAGTGATGAAGGTCTTCGGATCGTAAAGCTCTGT
TGTTAGGGAAGAACAAATGTGTAAGTAACTGTGCACATCTTGACGGTACCTAACCAGAAAGCCACGGCTAACTACGTGCCAGC
TGCCGCGGTAATACGTAGGTGGCAAGCGTTATCCGGAATTATTGGGCGTAAAGCGCGCGTAGGCGGTTTTTTAAGTCTGATGT
GAAAGCCCACGGCTCAACCGTGGAGGGTCATTGGAAACTGGAAAACTTGAGTGCAGAAGAGGAAAGTGGAATTCCATGTGTA
GCGGTGAAATGCGCAGAGATATGGAGGAACACCAGTGGCGAAGGCGGCTTTCTGGTCTGCAACTGACGCTGATGTGCGAAAG
CGTGGGGATCAAACA

>ASV13
TGGGGAATATTGCGCAATGGGGGGAACCCTGACGCAGCCATGCCGCGTGAATGAAGAAGGCCTTCGGGTTGTAAAGTTCTTT
CGGTAATGAGGAAGGGATGTTGTTAAATAGATAGCATCATTGACGTTAATTACAGAAGAAGCACCGGCTAACTCCGTGCCAGC
AGCCGCGGTAATACGGAGGGTGCGAGCGTTAATCGGAATAACTGGGCGTAAAGGGCACGCAGGCGGACTTTTAAGTGAGATG
TGAAATCCCCGAGCTTAACTTGGGAATTGCATTTCAGACTGGGAGTCTAGAGTACTTTAGGGAGGGGTAGAATTCCACGTGTA
GCGGTGAAATGCGTAGAGATGTGGAGGAATACCGAAGGCGAAGGCAGCCCCTTGGGAATGTACTGACGCTCATGTGCGAAAG
CGTGGGGAGCAAACA

>ASV14
TGGGGAATATTGCGCAATGGGGGGAACCCTGACGCAGCCATGCCGCGTGAATGAAGAAGGCCTTCGGGTTGTAAAGTTCTTT
CGGTAATGAGGAAGGGATGTTGTTAAATAGATAGCATCATTGACGTTAATTACAGAAGAAGCACCGGCTAACTCCGTGCCAGC
AGCCGCGGTAATACGGAGGGTGCGAGCGTTAATCGGAATAACTGGGCGTAAAGGGCACGCAGGCGGACTTTTAAGTGAGATG
TGAAATCCCCGAGCTTAACTTGGGAACTGCATTTCAGACTGGGAGTCTAGAGTACTTTAGGGAGGGGTAGAATTCCACGTGTA
GCGGTGAAATGCGTAGAGATGTGGAGGAATACCGAAGGCGAAGGCAGCCCCTTGGGAATGTACTGACGCTCATGTGCGAAAG
CGTGGGGAGCAAACA

>ASV15
TGGGGAATATTGGACAATGGGCGAAAGCCTGATCCAGCCATGCCGCGTGTGTGAAGAAGGTCTTCGGATTGTAAAGCACTTTA
AGTTGGGAGGAAGGGCAGTAAGTTAATACCTTGCTGTTTTGACGTTACCAACAGAATAAGCACCGGCTAACTTCGTGCCAGCT
GCCGCGGTAATACGAAGGGTGCAAGCGTTAATCGGAATTACTGGGCGTAAAGCGCGCGTAGGTGGTTCAGCAAGTTGGATGT
GAAATCCCCGGGCTCAACCTGGGAACTGCATCCAAAACTACTGAGCTAGAGTACGGTAGAGGGTGGTGGAATTTCCTGTGTAG
CGGTGAAATGCGTAGATATAGGAAGGAACACCAGTGGCGAAGGCGACCACCTGGACTGATACTGACACTGAGGTGCGAAAGC
GTGGGGAGCAAACA

>ASV16
TGGGGAATATTGCACAATGGGCGCAAGCCTGATGCAGCGACGCCGCGTGAGGGATGACGGCCTTCGGGTTGTAAACCTCTTT
CAGTAGGGAAGAAGCGAAAGTGACGGTACCTGCAGAAGAAGCACCGGCTAACTACGTGCCAGCAGCCGCGGTAATACGTAGG
GAGCGAGCGTTATCCGGAATTATTGGGCGTAAAGAGCTCGTAGGCGGTTTGTCGCGTCTGTCGTGAAAGTCCGGGGCTTAACC
CCGGATCTGCGGTGGGTACGGGCAGACTAGAGTGCAGTAGGGGAGACTGGAATTCCTGGTGTAGCGGTGGAATGCGCAGATA
TCAGGAGGAACACCGATGGCGAAGGCAGGTCTCTGGGCTGTAACTGACGCTGAGGAGCGAAAGCATGGGGAGCGAACA

>ASV17
TGGGGAATATTGCGCAATGGGGGGAACCCTGACGCAGCCATGCCGCGTGAATGAAGAAGGTCTTCGGGTTGTAAAGTTCTTTC
GGTAATGAGGAAGGGGTATTATTGAATAGATAATATCATTGACGTTAATTACAGAAGAAGCACCGGCTAACTCCGTGCCAGCA
GCCGCGGTAATACGGAGGGTGCGAGCGTTAATCGGAATAACTGGGCGTAAAGGGCACGCAGGCGGACTTTTAAGTGAGATGT
GAAATCCCCGAGCTTAACTTGGGAACTGCATTTCAGACTGGGAGTCTAGAGTACTTTAGGGAGGGGTAGAATTCCACGTGTAG
CGGTGAAATGCGTAGAGATGTGGAGGAATACCGAAGGCGAAGGCAGCCCCTTGGGAATGTACTGACGCTCATGTGCGAAAGC
GTGGGGAGCAAACA

>ASV18
TGGGGAATATTGCGCAATGGGGGGAACCCTGACGCAGCCATGCCGCGTGAATGAAGAAGGCCTTCGGGTTGTAAAGTTCTTT
CGGTGATGAGGAAGGGGTATTATTGAATAGATAATATCATTGACGTTAATTACAGAAGAAGCACCGGCTAACTCCGTGCCAGC
TGCCGCGGTAATACGGAGGGTGCGAGCGTTAATCGGAATAACTGGGCGTAAAGGGCACGCAGGCGGACTTTTAAGTGAGATG
TGAAATCCCCGAGCTTAACTTGGGAACTGCATTTCAGACTGGGAGTCTAGAGTACTTTAGGGAGGGGTAGAATTCCACGTGTA
GCGGTGAAATGCGTAGAGATGTGGAGGAATACCGAAGGCGAAGGCAGCCCCTTGGGAATGTACTGACGCTCATGTGCGAAAG
CGTGGGGAGCAAACA

>ASV19
TGGGGAATATTGCACAATGGGCGCAAGCCTGATGCAGCCATGCCGCGTGTATGAAGAAGGCCTTCGGGTTGTAAAGTACTTTC
AGCGGGGAGGAAGGGAGTAAAGTTAATACCTTTGCTCATTGACGTTACCCGCAGAAGAAGCACCGGCTAACTCCGTGCCAGC
TGCCGCGGTAATACGGAGGGTGCAAGCGTTAATCGGAATTACTGGGCGTAAAGCGCACGCAGGCGGTTTGTTAAGTCAGATG
TGAAATCCCCGGGCTCAACCTGGGAACTGCATCTGATACTGGCAAGCTTGAGTCTCGTAGAGGGGGGTAGAATTCCAGGTGTA
GCGGTGAAATGCGTAGAGATCTGGAGGAATACCGGTGGCGAAGGCGGCCCCCTGGACGAAGACTGACGCTCAGGTGCGAAAG
CGTGGGGAGCAAACA

>ASV20
TGGGGAATATTGGACAATGGGCGCAAGCCTGATCCAGCCATGCCGCGTGTGTGAAGAAGGCCTTATGGTTGTAAAGCACTTTA
AGCGAGGAGGAGGCTACTTTAGATAATACCTAGAGATAGTGGACGTTACTCGCAGAATAAGCACCGGCTAACTCTGTGCCAGT
AGCCGCGGTAATACAGAGGGTGCAAGCGTTAATCGGATTTACTGGGCGTAAAGCGCGCGTAGGCGGCTAATTAAGTCAAATG
TGAAATCCCCGAGCTTAACTTGGGAATTGCATTCGATACTGGTTAGCTAGAGTGTGGGAGAGGATGGTAGAATTCCAGGTGTA
GCGGTGAAATGCGTAGAGATCTGGAGGAATACCGATGGCGAAGGCAGCCATCTGGCCTAACACTGACGCTGAGGTGCGAAAG
CATGGGGAGCAAACA

>ASV21
TGGGGAATATTGCACAATGGGCGCAAGCCTGATGCAGCCATGCCGCGTGTATGAAGAAGGCCTTCGGGTTGTAAAGTACTTTC
AGCGGGGAGGAAGGGATGGAGCTTAATACGCTTCGTCATTGACGTTACCCGCAGAAGAAGCACCGGCTAACTCCGTGCCAGC
AGCCGCGGTAATACGGAGGGTGCAAGCGTTAATCGGAATTACTGGGCGTAAAGCGCACGCAGGCGGTCTGTCAAGTCGGATG
TGAAATCCCCGGGCTTAACCTGGGAACTGCATTCGAAACTGGCAGGCTAGAGTCTTGTAGAGGGGGGTAGAATTCCAGGTGTA
GCGGTGAAATGCGTAGAGATCTGGAGGAATACCGGTGGCGAAGGCGGCCCCCTGGACAAAGACTGACGCTCAGGTGCGAAAG
CGTGGGGAGCAAACA

>ASV22

TAGGGAATCTTCCGCAATGGACGAAAGTCTGACGGAGCAACGCCGCGTGAGTGATGAAGGCTTTCGGGTCGTAAAACTCTGTT
GTTAGGGAAGAACAAGTGCTAGTTGAATAAGCTGGCACCTTGACGGTACCTAACCAGAAAGCCACGGCTAACTACGTGCCAG
CAGCCGCGGTAATACGTAGGTGGCAAGCGTTATCCGGAATTATTGGGCGTAAAGCGCGCGCAGGTGGTTTCTTAAGTCTGATG
TGAAAGCCCACGGCTCAACCGTGGAGGGTCATTGGAAACTGGGAGACTTGAGTGCAGAAGAGGAAAGTGGAATTCCATGTGT
AGCGGTGAAATGCGTAGAGATATGGAGGAACACCAGTGGCGAAGGCGACTTTCTGGTCTGTAACTGACGCTGATGTGCGAAA
GCGTGGGGATCAAACA

>ASV23
TAGGGAATCTTCCGCAATGGGCGAAAGCCTGACGGAGCAACGCCGCGTGAGTGATGAAGGTCTTCGGATCGTAAAACTCTGT
TATTAGGGAAGAACAAATGTGTAAGTAACTATGCACGTCTTGACGGTACCTAATCAGAAAGCCACGGCTAACTACGTGCCAGC
AGCCGCGGTAATACGTAGGTGGCAAGCGTTATCCGGAATTATTGGGCGTAAAGCGCGCGCAGGTGGTTTCTTAAGTCTGATGT
GAAAGCCCACGGCTCAACCGTGGAGGGTCATTGGAAACTGGGAGACTTGAGTGCAGAAGAGGAAAGTGGAATTCCATGTGTA
GCGGTGAAATGCGTAGAGATATGGAGGAACACCAGTGGCGAAGGCGACTTTCTGGTCTGTAACTGACACTGAGGCGCGAAAG
CGTGGGGAGCAAACA

>ASV24
TGGGGAATATTGCGCAATGGGGGGAACCCTGACGCAGCCATGCCGCGTGAATGAAGAAGGCCTTCGGGTTGTAAAGTTCTTT
CGGTAATGAGGAAGGGATGTTGTTTAATAGATAGCATCATTGACGTTAATTACAGAAGAAGCACCGGCTAACTCCGTGCCAGC
TGCCGCGGTAATACGGAGGGTGCGAGCGTTAATCGGAATAACTGGGCGTAAAGGGCACGCAGGCGGACTTTTAAGTGAGATG
TGAAATCCCCGAGCTTAACTTGGGAATTGCATTTCAGACTGGGAGTCTAGAGTACTTTAGGGAGGGGTAGAATTCCACGTGTA
GCGGTGAAATGCGTAGAGATGTGGAGGAATACCGAAGGCGAAGGCAGCCCCTTGGGAATGTACTGACGCTCATGTGCGAAAG
CGTGGGGAGCAAACA

>ASV25
TAGGGAATCTTCGGCAATGGACGAAAGTCTGACCGAGCAACGCCGCGTGAGTGAAGAAGGTTTTCGGATCGTAAAACTCTGTT
GTTAGAGAAGAACAAGGATGAGAGTAACTGTTCATCCCTTGACGGTATCTAACCAGAAAGCCACGGCTAACTACGTGCCAGCA
GCCGCGGTAATACGTAGGTGGCAAGCGTTATCCGGAATTATTGGGCGTAAAGCGCGCGTAGGCGGTTTTTTAAGTCTGATGTG
AAAGCCCACGGCTCAACCGTGGAGGGTCATTGGAAACTGGAAAACTTGAGTGCAGAAGAGGAAAGTGGAATTCCATGTGTAG
CGGTGAAATGCGCAGAGATATGGAGGAACACCAGTGGCGAAGGCGGCTTTCTGGTCTGCAACTGACGCTGATGTGCGAAAGC
GTGGGGATCAAACA

>ASV26
TAGGGAATCTTCCGCAATGGGCGAAAGCCTGACGGAGCAACGCCGCGTGAGTGATGAAGGTCTTCGGATCGTAAAGCTCTGT
TGTTAGGGAAGAACAAATGTGTAAGTAACTGTGCACATCTTGACGGTACCTAACCAGAAAGCCACGGCTAACTACGTGCCAGC
AGCCGCGGTAATACGTAGGTGGCAAGCGTTATCCGGAATTATTGGGCGTAAAGCGCGTAGGCGGTTTTTTAAGTCTGATGT
GAAAGCCCACGGCTCAACCGTGGAGGGTCATTGGAAACTGGAAAACTTGAGTGCAGAAGAGGAAAGTGGAATTCCATGTGTA
GCGGTGAAATGCGCAGAGATATGGAGGAACACCAGTGGCGAAGGCGGCTCTCTGGTCTGTAACTGACGCTGAGGCTCGAAAG
CGTGGGGAGCAAACA

>ASV27
TAGGGAATCTTCCGCAATGGACGAAAGTCTGACGGAGCAACGCCGCGTGAGTGATGAAGGCTTTCGGGTCGTAAAACTCTGTT
GTTAGGGAAGAACAAGTGCTAGTTGAATAAGCTGGCACCTTGACGGTACCTAACCAGAAAGCCACGGCTAACTACGTGCCAG
CAGCCGCGGTAATACGTAGGTGGCAAGCGTTATCCGGAATTATTGGGCGTAAAGCGCGCGTAGGCGGTTTTTTAAGTCTGATG
TGAAAGCCCACGGCTCAACCGTGGAGGGTCATTGGAAACTGGAAAACTTGAGTGCAGAAGAGGAAAGTGGAATTCCATGTGT
AGCGGTGAAATGCGCAGAGATATGGAGGAACACCAGTGGCGAAGGCGACTTTCTGGTCTGTAACTGACGCTGATGTGCGAAA
GCGTGGGGATCAAACA

>ASV28
TAGGGAATCTTCGGCAATGGACGAAAGTCTGACCGAGCAACGCCGCGTGAGTGAAGAAGGTTTTCGGATCGTAAAACTCTGTT
GTTAGAGAAGAACAAGGATGAGAGTAACTGTTCATCCCTTGACGGTATCTAACCAGAAAGCCACGGCTAACTACGTGCCAGCA
GCCGCGGTAATACGTAGGTGGCAAGCGTTGTCCGGATTTATTGGGCGTAAAGCGAGCGCAGGCGGTTTCTTAAGTCTGATGTG
AAAGCCCCCGGCTCAACCGGGGAGGGTCATTGGAAACTGGGAGACTTGAGTGCAGAAGAGGAGAGTGGAATTCCATGTGTAG
CGGTGAAATGCGTAGATATATGGAGGAACACCAGTGGCGAAGGCGGCTCTCTGGTCTGTAACTGACGCTGAGGCTCGAAAGC
GTGGGGAGCAAACA

>ASV29

TGGGGAATATTGGACAATGGGCGAAAGCCTGATCCAGCCATGTCGCGTGTGTGAAGAAGGTCTTCGGATTGTAAAGCACTTTA
AGTTGGGAGGAAGGGCAGTAAGTTAATACCTTGCTGTTTTGACGTTACCAACAGAATAAGCACCGGCTAACTTCGTGCCAGCA
GCCGCGGTAATACGAAGGGTGCAAGCGTTAATCGGAATTACTGGGCGTAAAGCGCGCGTAGGTGGTTCAGCAAGTTGGATGT
GAAATCCCCGGGCTCAACCTGGGAACTGCATCCAAAACTACTGAGCTAGAGTACGGTAGAGGGTGGTGGAATTTCCTGTGTAG
CGGTGAAATGCGTAGATATAGGAAGGAACACCAGTGGCGAAGGCGACCACCTGGACTGATACTGACACTGAGGTGCGAAAGC
GTGGGGAGCAAACA

>ASV30
TAGGGAATCTTCCGCAATGGGCGAAAGCCTGACGGAGCAACGCCGCGTGAGTGATGAAGGTCTTAGGATCGTAAAACTCTGT
TATTAGGGAAGAACAAACGTGTAAGTAACTGTGCACGTCTTGACGGTACCTAATCAGAAAGCCACGGCTAACTACGTGCCAGC
AGCCGCGGTAATACGTAGGTGGCAAGCGTTATCCGGAATTATTGGGCGTAAAGCGCGCGCAGGTGGTTTCTTAAGTCTGATGT
GAAAGCCCACGGCTCAACCGTGGAGGGTCATTGGAAACTGGGAGACTTGAGTGCAGAAGAGGAAAGTGGAATTCCATGTGTA
GCGGTGAAATGCGTAGAGATATGGAGGAACACCAGTGGCGAAGGCGACTTTCTGGTCTGTAACTGACACTGAGGCGCGAAAG
CGTGGGGAGCAAACA

>ASV31
TGGGGAATATTGGACAATGGGCGAAAGCCTGATCCAGCCATGCCGCGTGTGTGAAGAAGGTCTTCGGATTGTAAAGCACTTTA
AGTTGGGAGGAAGGGCAGTAAGTTAATACCTTGCTGTTTTGACGTTACCAACAGAATAAGCACCGACTAACTTCGTGCCAGCA
GCCGCGGTAATACGAAGGGTGCAAGCGTTAATCGGAATTACTGGGCGTAAAGCGCGCGTAGGTGGTTCAGCAAGTTGGATGT
GAAATCCCCGGGCTCAACCTGGGAACTGCATCCAAAACTACTGAGCTAGAGTACGGTAGAGGGTGGTGGAATTTCCTGTGTAG
CGGTGAAATGCGTAGATATAGGAAGGAACACCAGTGGCGAAGGCGACCACCTGGACTGATACTGACACTGAGGTGCGAAAGC
GTGGGGAGCAAACA

>ASV32
TGGGGAATATTGCACAATGGGCGCAAGCCTGATGCAGCCATGCCGCGTGTATGAAGAAGGCCTTCGGGTTGTAAAGTACTTTC
AGCGGGGAGGAAGGGATGAAGCTTAATACGCTTTGTCATTGACGTTACCCGCAGAAGAAGCACCGGCTAACTCCGTGCCAGC
AGCCGCGGTAATACGGAGGGTGCAAGCGTTAATCGGAATTACTGGGCGTAAAGCGCACGCAGGCGGTCTGTCAAGTCGGATG
TGAAATCCCCGGGCTTAACCTGGGAACTGCATTCGAAACTGGCAGGCTAGAGTCTTGTAGAGGGGGGTAGAATTCCAGGTGTA
GCGGTGAAATGCGTAGAGATCTGGAGGAATACCGGTGGCGAAGGCGGCCCCCTGGACAAAGACTGACGCTCAGGTGCGAAAG
CGTGGGGAGCAAACA

>ASV33
TAGGGAATCTTCCGCAATGGACGAAAGCCTGACGGAGCAACGCCGCGTGAGTGATGAAGGTCTTCGGATCGTAAAGCTCTGT
TGTTAGGGAAGAACAAATGTGTAAGTAACTGTGCACATCTTGACGGTACCTAACCAGAAAGCCACGGCTAACTACGTGCCAGC
AGCCGCGGTAATACGTAGGTGGCAAGCGTTATCCGGAATTATTGGGCGTAAAGCGCGCGTAGGCGGTTTTTTAAGTCTGATGT
GAAAGCCCACGGCTCAACCGTGGAGGGTCATTGGAAACTGGAAAACTTGAGTGCAGAAGAGGAAAGTGGAATTCCATGTGTA
GCGGTGAAATGCGCAGAGATATGGAGGAACACCAGTGGCGAAGGCGGCTTTCTGGTCTGCAACTGACGCTGATGTGCGAAAG
CGTGGGGATCAAACA

>ASV34
TAGGGAATCTTCGGCAATGGACGAAAGTCTGACCGAGCAACGCCGCGTGAGTGAAGAAGGTTTTCGGATCGTAAAGCTCTGTT
GTAAGTCAAGAACGTGTGTGAGAGTGGAAAGTTCACACAGTGACGGTAGCTTACCAGAAAGGGACGGCTAACTACGTGCCAG
CAGCCGCGGTAATACGTAGGTCCCGAGCGTTGTCCGGATTTATTGGGCGTAAAGGGAGCGCAGGCGGTCAGGAAAGTCTGGA
GTAAAAGGCTATGGCTCAACCATAGTGTGCTCTGGAAACTGTCTGACTTGAGTGCAGAAGGGGAGAGTGGAATTCCATGTGTA
GCGGTGAAATGCGTAGATATATGGAGGAACACCAGTGGCGAAAGCGGCTCTCTGGTCTGTCACTGACGCTGAGGCTCGAAAG
CGTGGGTAGCGAACA

>ASV35
TGGGGAATATTGGACAATGGGCGCAAGCCTGATCCAGCCATGCCGCGTGAGTGATGAAGGCCCTAGGGTTGTAAAGCTCTTTC
ACCGGTGAAGATAATGACGGTAACCGGAGAAGAAGCCCCGGCTAACTTCGTGCCAGCAGCCGCGGTAATACGAAGGGGGCTA
GCGTTGTTCGGATTTACTGGGCGTAAAGCGCACGTAGGCGGACTATTAAGTCAGGGGTGAAATCCCGGGGCTCAACCCCGGA
ACTGCCTTTGATACTGGTAGTCTTGAGTTCGAGAGAGGTGAGTGGAATTCCGAGTGTAGAGGTGAAATTCGTAGATATTCGGA
GGAACACCAGTGGCGAAGGCGGCTCACTGGCTCGATACTGACGCTGAGGTGCGAAAGCGTGGGGAGCAAACA

>ASV36
TGGGGAATATTGCACAATGGGCGCAAGCCTGATGCAGCGACGCCGCGTGAGGGATGACGGCCTTCGGGTTGTAAACCTCTTT
CAGTAGGGAAGAAGCGAAAGTGACGGTACCTGCAGAAGAAGCACCGGCTAACTACGTGCCAGCAGCCGCGGTAATACGTAGG

GTGCGAGCGTTATCCGGAATTATTGGGCGTAAAGAGCTCGTAGGCGGTTTGTCGCGTCTGTCGTGAAAGTCCGGGGCTTAACC
CCGGATCTGCGGTGGGTACGGGCAGACTAGAGTGCAGTAGGGGAGACTGGAATTCCTGGTGTAGCGGTGGAATGTGCAGATA
TCAGGAGGAACACCGATGGCGAAGGCAGGTCTCTGGGCTGTAACTGACGCTGAGGAGCGAAAGCATGGGGAGCGAACA

>ASV37
TAGGGAATCTTCCGCAATGGGCGAAAGCTTGACGGAGCAACGCCGCGTGAGTGATGAAGGTCTTCGGATCGTAAAACTCTGTT
ATTAGGGAAGAACAAATGTGTAAGTAACTATGCACGTCTTGACGGTACCTAATCAGAAAGCCACGGCTAACTACGTGCCAGCA
GCCGCGGTAATACGTAGGTGGCAAGCGTTATCCGGAATTATTGGGCGTAAAGCGCGCGCAGGTGGTTTCTTAAGTCTGATGTG
AAAGCCCACGGCTCAACCGTGGAGGGTCATTGGAAACTGGGAGACTTGAGTGCAGAAGAGGAAAGTGGAATTCCATGTGTAG
CGGTGAAATGCGTAGAGATATGGAGGAACACCAGTGGCGAAGGCGACTTTCTGGTCTGTAACTGACACTGAGGCGCGAAAGC
GTGGGGAGCAAACA

>ASV38
TGGGGAATATTGCACAATGGGCGCAAGCCTGATGCAGCCATGCCGCGTGTATGAAGAAGGCCTTCGGGTTGTAAAGTACTTTC
AGCGGGGAGGAAGGGAGTAAAGTTAATACCTTTGCTCATTGACGTTACCCGCAGAAGAAGCACCGGCTAACTCCGTGCCAGC
AGCCGCGGTAATACGGAGGGTGCAAGCGTTAATCGGAATTACTGGGCGTAAAGCGCACGCAGGCGGTTTGTTAAGTCAGATG
TGAAATCCCCGGGCTCAACCTGGGAACTGCATCTGATACTGGCAAGCTTGAGTCTCGTAGAGGGGGGTAGAATTCCAGGTGTA
GCGGTGAAATGCGTAGAGATCTGGAGGAATACCGGTGGCGAAGGTGGCCCCCTGGACGAAGACTGACGCTCAGGTGCGAAAG
CGTGGGGAGCAAACA

>ASV39
TAAGGAATATTGGACAATGGACGCAAGTCTGATCCAGCCATGCCGCGTGAAGGATGAATGCCCTCTGGGTTGTAAACTTCTTT
TACAGGGGAAGAAAGTTATCTTTTTCAGGATATTTGACGGTACCCTATGAATAAGCACCGGCTAACTCCGTGCCAGCAGCCGC
GGTAATACGGAGGGTGCAAGCGTTATCCGGATTCACTGGGTTTAAAGGGTGCGTAGGCGGGTATGTAAGTCAGTGGTGAAATA
CCGGAGCTTAACTTCGGAACTGCCATTGATACTATATATCTTGAATATTGTGGAGGTAAGCGGAATATGTCATGTAGCGGTGA
AATGCTTAGAGATGACATAGAACACCGATTGCGAAGGCAGCTTGCTACGCAAATATTGACGCTGAGGCACGAAAGCGTGGGG
ATCAAACA

>ASV40
TGGGGAATATTGGACAATGGGCGCAAGCCTGATCCAGCCATGCCGCGTGAGTGATGAAGGCCCTAGGGTTGTAAAGCTCTTTC
AACGGTGAAGATAATGACGGTAACCGTAGAAGAAGCCCCGGCTAACTTCGTGCCAGCAGCCGCGGTAATACGAAGGGGGCTA
GCGTTGTTCGGATTTACTGGGCGTAAAGCGCACGTAGGCGGATTGTTAAGTTAGGGGTGAAATCCCAGGGCTCAACCCTGGAA
CTGCCTTTAATACTGGCAATCTCGAGTCCGGAAGAGGTGAGTGGAATTCCGAGTGTAGAGGTGAAATTCGTAGATATTCGGAG
GAACACCAGTGGCGAAGGCGGCTCACTGGTCCGGTACTGACGCTGAGGTGCGAAAGCGTGGGGAGCAAACA

>ASV41
TAGGGAATCTTCCGCAATGGGCGAAAGCCTGACGGAGCAACGCCGCGTGAGTGATGAAGGTCTTAGGATCGTAAAACTCTGT
TATTAGGGAAGAACAAACGTGTAAGTAACTGTGCACGTCTTGACGGTACCTAATCAGAAAGCCACGGCTAACTACGTGCCAGC
AGCCGCGGTAATACGTAGGTGGCAAGCGTTATCCGGAATTATTGGGCGTAAAGCGCGCGTAGGCGGTTTTTTAAGTCTGATGT
GAAAGCCCACGGCTCAACCGTGGAGGGTCATTGGAAACTGGAAAACTTGAGTGCAGAAGAGGAAAGTGGAATTCCATGTGTA
GCGGTGAAATGCGCAGAGATATGGAGGAATACCAGTGGCGAAGGCGACTTTCTGGTCTGTAACTGACGCTGATGTGCGAAAG
CGTGGGGATCAAACA

>ASV42
TAGGGAATCTTCCGCAATGGGCGAAAGCCTGACGGAGCAACGCCGCGTGAGTGATGAAGGTCTTCGGATCGTAAAGCTCTGT
TGTTAGGGAAGAACAAATGTGTAAGTAACTGTGCACATCTTGACGGTACCTAACCAGAAAGCCACGGCTAACTACGTGCCAGC
AGCCGCGGTAATACGTAGGGTGCGAGCGTTAATCGGAATTACTGGGCGTAAAGCGGGCGCAGACGGTTACTTAAGTCAGATG
TGAAATCCCCGGGCTCAACCTGGGAACTGCGTTTGAAACTGGGTGACTAGAGTATGTCAGAGGGGGGTAGAATTCCACGTGTA
GCAGTGAAATGCGTAGAGATGTGGAGGAATACCGATGGCGAAGGCAGCCCCCTGGGATAATACTGACGTTCATGCCCGAAAG
CGTGGGTAGCAAACA

>ASV43
TAGGGAATCTTCGGCAATGGACGAAAGTCTGACCGAGCAACGCCGCGTGAGTGAAGAAGGTTTTCGGATCGTAAAACTCTGTT
GTTAGAGAAGAATAAGGATGAGAGTAACTGTTCATCCCTTGACGGTATCTAACCAGAAAGCCACGGCTAACTACGTGCCAGCA
GCCGCGGTAATACGTAGGTGGCAAGCGTTGTCCGGATTTATTGGGCGTAAAGCGAGCGCAGGCGGTTTCTTAAGTCTGATGTG
AAAGCCCCCGGCTCAACCGGGGAGGGTCATTGGAAACTGGGAGACTTGAGTGCAGAAGAGGAGAGTGGAATTCCATGTGTAG

CGGTGAAATGCGTAGATATATGGAGGAACACCAGTGGCGAAGGCGGCTCTCTGGTCTGCAACTGACGCTGAGGCTCGAAAGC
GTGGGGAGCAAACA

>ASV44
TGGGGAATTTTGGACAATGGGGGGAACCCTGATCCAGCCATGCCGCGTGTCTGAAGAAGGCCTTCGGGTTGTAAAGGACTTTT
GTCAGGGAAGAAAAGCTTGAGGTTAATACCCTTGAGTGATGACGGTACCTGAAGAATAAGCACCGGCTAACTACGTGCCAGC
AGCCGCGGTAATACGTAGGGTGCGAGCGTTAATCGGAATTACTGGGCGTAAAGCGGGCGCAGACGGTTACTTAAGTCAGATG
TGAAATCCCCGGGCTCAACCTGGGAACTGCGTTTGAAACTGGGTGACTAGAGTATGTCAGAGGGGGGTAGAATTCCACGTGTA
GCAGTGAAATGCGTAGAGATGTGGAGGAATACCGATGGCGAAGGCAGCCCCCTGGGATAATACTGACGTTCATGCCCGAAAG
CGTGGGTAGCAAACA

>ASV45
TGGGGAATATTGGACAATGGGCGAAAGCCCGATCCAGCAATATCGCGTGAGTGAAGAAGGGCAATGCCGCTTGTAAAGCTCT
TTCGTCGAGTGCGCGATCATGACAGGACTCGAGGAAGAAGCCCCGGCTAACTCCGTGCCAGCAGCCGCGGTAAGACGGGGG
GGCAAGTGTTCTTCGGAATGACTGGGCGTAAAGGGCACGTAGGCGGTGAATCGGGTTGAAAGTGAAAGTCGCCAAAAAGTGG
CGGAATGCTCTCGAAACCAATTCACTTGAGTGAGACAGAGGAGAGTGGAATTTCGTGTGTAGGGGTGAAATCCGTAGATCTAC
GAAGGAACGCCAAAAGCGAAGGCAGCTCTCTGGGTCCCTACCGACGCTGGGGTGCGAAAGCATGGGGAGCGAACA

>ASV46
TGGGGAATTTTCCGCAATGGGCGAAAGCCTGACGGAGCAATGCCGCGTGGAGGTGGAAGGCCTACGGGTCGTCAACTTCTTT
TCTCGGAGAAGAAACAATGACGGTATCTGAGGAATAAGCATCGGCTAACTCTGTGCCAGCAGCCGCGGTAAGACAGAGGATG
CAAGCGTTATCCGGAATGATTGGGCGTAAAGCGTCTGTAGGTGGCTTTTCAAGTCCGCCGTCAAATCCCAGGGCTCAACCCTG
GACAGGCGGTGGAAACTACCAAGCTGGAGTACGGTAGGGGCAGAGGGAATTTCCGGTGGAGCGGTGAAATGCATTGAGATCG
GAAAGAACACCAACGGCGAAAGCACTCTGCTGGGCCGACACTGACACTGAGAGACGAAAGCTAGGGGAGCAAATG

>ASV47
TGGGGAATATTGCACAATGGGCGAAAGCCTGATGCAGCGACGCCGCGTGAGGGATGACGGCCTTCGGGTTGTAAACCTCTTT
CAGTAGGGAAGAAGCGAAAGTGACGGTACCTGCAGAAGAAGCACCGGCTAACTACGTGCCAGCAGCCGCGGTAATACGTAGG
GTGCGAGCGTTATCCGGAATTATTGGGCGTAAAGAGCTCGTAGGCGGTTTGTCGCGTCTGTCGTGAAAGTCCGGGGCTTAACC
CCGGATCTGCGGTGGGTACGGGCAGACTAGAGTGCAGTAGGGGAGACTGGAATTCCTGGTGTAGCGGTGGAATGCGCAGATA
TCAGGAGGAACACCGATGGCGAAGGCAGGTCTCTGGGCTGTAACTGACGCTGAGGAGCGAAAGCATGGGGAGCGAACA

>ASV48
TGGGGAATTTTCCGCAATGGGCGAAAGCCTGACGGAGCAATGCCGCGTGGAGGTAGAAGGCCTACGGGTCGTGAACTTCTTT
TCCCGGAGAAGAAGCAATGACGGTATCTGGGGAATAAGCATCGGCTAACTCTGTGCCAGCAGCCGCGGTAATACAGAGGATG
CAAGCGTTATCCGGAATGATTGGGCGTAAAGCGTCTGTAGGTGGCTTTTTAAGTCCGCCGTCAAATCCCAGGGCTCAACCCTG
GACAGGCGGTGGAAACTACCAAGCTGGAGTACGGTAGGGGCAGAGGGAATTTCCGGTGGAGCGGTGAAATGCGTAGAGATCG
GAAAGAACACCAACGGCGAAAGCACTCTGCTGGGCCGACACTGACACTGAGAGACGAAAGCTAGGGGAGCGAATG

>ASV49
TAGGGAATCTTCCGCAATGGACGAAAGTCTGACGGAGCAACGCCGCGTGAGTGATGAAGGTTTTCGGATCGTAAAGCTCTGTT
GTTAGGGAAGAACAAGTGCCGTTCAAATAGGGCGGCACCTTGACGGTACCTAACCAGAAAGCCACGGCTAACTACGTGCCAG
CAGCCGCGGTAATACGTAGGTGGCAAGCGTTGTCCGGAATTATTGGGCGTAAAGGGCTCGCAGGCGGTTTCTTAAGTCTGATG
TGAAAGCCCCCGGCTCAACCGGGGAGGGTCATTGGAAACTGGGGAACTTGAGTGCAGAAGAGGAGAGTGGAATTCCACGTGT
AGCGGTGAAATGCGTAGAGATGTGGAGGAACACCAGTGGCGAAGGCGACTCTCTGGTCTGTAACTGACGCTGAGGAGCGAAA
GCGTGGGGAGCGAACA

>ASV50
TGGGGAATATTGCACAATGGGCGCAAGCCTGATGCAGCCATGCCGCGTGTATGAAGAAGGCCTTCGGGTTGTAAAGTACTTTC
AGCGGGGAGGAAGGGAGTAAAGTTAATACCTTTGCTCATTGACGTTACCCGCAGAAGAAGCACCGGCTAACTTCGTGCCAGC
AGCCGCGGTAATACGAAGGGTGCAAGCGTTAATCGGAATTACTGGGCGTAAAGCGCGCGTAGGTGGTTCAGCAAGTTGGATG
TGAAATCCCCGGGCTCAACCTGGGAACTGCATCCAAAACTACTGAGCTAGAGTACGGTAGAGGGTGGTGGAATTTCCTGTGTA
GCGGTGAAATGCGTAGATATAGGAAGGAACACCAGTGGCGAAGGCGACCACCTGGACTGATACTGACACTGAGGTGCGAAAG
CGTGGGGAGCAAACA

>ASV51

TGGGGAATATTGCACAATGGGCGCAAGCCTGATGCAGCGACGCCGCGTGAGGGATGACGGCCTTCGGGTTGTAAACCTCTTT
CAGTAGGGAAGAAGCGAAAGTGACGGTACCTGCAGAAGAAGCACCGGCTAACTACGTGCCAGCAGCCGCGGTAATACGTAGG
TGGCAAGCGTTATCCGGAATTATTGGGCGTAAAGCGCGCGTAGGCGGTTTTTTAAGTCTGATGTGAAAGCCCACGGCTCAACC
GTGGAGGGTCATTGGAAACTGGAAAACTTGAGTGCAGAAGAGGAAAGTGGAATTCCATGTGTAGCGGTGAAATGCGCAGAGA
TATGGAGGAACACCAGTGGCGAAGGCGGCTTTCTGGTCTGCAACTGACGCTGATGTGCGAAAGCGTGGGGATCAAACA

>ASV52
TAGGGAATATTGCTCAATGGGGGAAACCCTGAAGCAGCAACGCCGCGTGGAGGATGACACTTTTCGGAGCGTAAACTCCTTTT
CTTGGGAAAGAATTATGACGGTACCCAAGGAATAAGCACCGGCTAACTCCGTGCCAGCAGCCGCGGTAATACGGAGGGTGCA
AGCGTTACTCGGAATCACTGGGCGTAAAGGACGCGTAGGCGGATTATCAAGTCTTGAGTGAAATCTAACGGCTTAACCGTTAA
ACTGCTTGGGAAACTGATAATCTAGAGTAAGGGAGAGGCAGATGGAATTCTTGGTGTAGGGGTAAAATCCGTAGAGATCAAGA
AGAATACCCATTGCGAAGGCGATCTGCTGGAACTTAACTGACGCTAATGCGTGAAAGCGTGGGGAGCAAACA

>ASV53
TGGGGAATATTGGACAATGGGCGCAAGCCTGATCCAGCCATGCCGCGTGAGTGATGAAGGCCCTAGGGTTGTAAAGCTCTTTT
GTGCGGGAAGATAATGACGGTACCGCAAGAATAAGCCCCGGCTAACTTCGTGCCAGCAGCCGCGGTAATACGAAGGGGGCTA
GCGTTGCTCGGAATCACTGGGCGTAAAGGGTGCGTAGGCGGGTCTTTAAGTCAGGGGTGAAATCCTGGAGCTCAACTCCAGA
ACTGCCTTTGATACTGAAGATCTTGAGTTCGGGAGAGGTGAGTGGAACTGCGAGTGTAGAGGTGAAATTCGTAGATATTCGCA
AGAACACCAGTGGCGAAGGCGGCTCACTGGCCCGATACTGACGCTGAGGCACGAAAGCGTGGGGAGCAAACA

>ASV54
TGGGGAATATTGGACAATGGGCGCAAGCCTGATCCAGCCATGCCGCGTGAGTGATGACGGCCTTAGGGTTGTAAAGCTCTTTC
GACGGGGACGATAATGACGGTACCCGTAGAAGAAGCCCCGGCTAACTTCGTGCCAGCAGCCGCGGTAATACGAAGGGGGCTA
GCGTTGTTCGGAATCACTGGGCGTAAAGCGCACGTAGGCGGATTGTTAAGTCGGGGGTGAAATCCTGAGGCTCAACCTCAGA
ACTGCCTTCGATACTGGCAATCTCGAGTCCGGAAGAGGTTGGTGGAACAGCTAGTGTAGAGGTGAAATTCGTAGATATTAGCT
AGAACACCAGTGGCGAAGGCGGCCAACTGGTCCGGCACTGACGCTGAGGTGCGAAAGCGTGGGGAGCAAACA

>ASV55
TAAGGAATATTGGTCAATGGACGGAAGTCTGAACCAGCCATGCCGCGTGGAGGATGAAGGTCCTCTGGATTGTAAACTTCTTT
TATTTGGGACGAAATGATCTTTTTCTAAAGGTTTTGACGGTACCAGATGAATAAGCACCGGCTAACTCCGTGCCAGCAGCCGT
GGTAATACGGAGGGTGCAAGCGTTATCCGGATTCACTGGGTTTAAAGGGTGCGTAGGCGGACACTTAAGTCCGTGGTGAAATC
TCCGGGCTTAACCCGGAAACTGCCATGGATACTATTTGTCTTGAATGCTGTGGAGGTTAGCGGAATATGTCATGTAGCGGTGA
AATGCATAGATATGACATAGAACACCAATTGCGAAGGCAGCTGGCTACACAGAAATTGACGCTGAGGCACGAAAGCGTGGGG
ATCAAACA

>ASV56
TGGGGAATTTTGGACAATGGGCGAAAGCCTGATCCAGCCATGCCGCGTGCAGGATGAAGGCCTTCGGGTTGTAAACTGCTTTT
GTACGGAACGAAACGGCCTTTTCTAATAAAGAGGGCTAATGACGGTACCGTAAGAATAAGCACCGGCTAACTACGTGCCAGC
AGCCGCGGTAATACGTAGGGTGCAAGCGTTAATCGGAATTACTGGGCGTAAAGCGTGCGCAGGCGGTTATGTAAGACAGTTG
TGAAATCCCCGGGCTCAACCTGGGAACTGCATCTGTGACTGCATAGCTAGAGTACGGTAGAGGGGGATGGAATTCCGCGTGT
AGCAGTGAAATGCGTAGATATGCGGAGGAACACCGATGGCGAAGGCAATCCCCTGGACCTGTACTGACGCTCATGCACGAAA
GCGTGGGGAGCAAACA

>ASV57
TGGGGAATATTGGACAATGGGCGAAAGCCTGATCCAGCCATGCCGCGTGTGTGAAGAAGGTCTTCGGATCGTAAAGCTCTGTT
GTTAGGGAAGAACAAATGTGTAAGTAACTGTGTACATCTTGACGGTACCTAACCAGAAAGCCACGGCTAACTACGTGCCAGCA
GCCGCGGTAATACGTAGGTGGCAAGCGTTATCCGGAATTATTGGGCGTAAAGCGCGCGTAGGCGGTTTTTTAAGTCTGATGTG
AAAGCCCACGGCTCAACCGTGGAGGGTCATTGGAAACTGGAAAACTTGAGTGCAGAAGAGGAAAGTGGAATTCCATGTGTAG
CGGTGAAATGCGCAGAGATATGGAGGAACACCAGTGGCGAAGGCGGCTTTCTGGTCTGCAACTGACGCTGATGTGCGAAAGC
GTGGGGATCAAACA

>ASV58
TGGGGAATATTGCACAATGGGCGCAAGCCTGATGCAGCCATGCCGCGTGTATGAAGAAGGCCTTCGGGTTGTAAAGTACTTTC
AGCGGGGAGGAAGGCGACGGGGTTAATAACCCTGTCGATTGACGTTACCCGCAGAAGAAGCACCGGCTAACTCCGTGCCAGC
AGCCGCGGTAATACGGAGGGTGCAAGCGTTAATCGGAATTACTGGGCGTAAAGCGCACGCAGGCGGTCTGTTAAGTCAGATG
TGAAATCCCCGGGCTTAACCTGGGAACTGCATTTGAAACTGGCAGGCTTGAGTCTTGTAGAGGGGGGTAGAATTCCAGGTGTA

GCGGTGAAATGCGTAGAGATCTGGAGGAATACCGGTGGCGAAGGCGGCCCCCTGGACAAAGACTGACGCTCAGGTGCGAAAG
CGTGGGGAGCAAACA

>ASV59
TGGGGAATATTGGACAATGGGCGAAAGCCTGATCCAGCCATGCCGCGTGTGTGAAGAAGGTCTTCGGATTGTAAAGCACTTTA
AGTTGGGAGGAAGGGCAGTAAGTTAATACCTTGCTGTTTTGACGTTACCAACAGAATAAGCACCGGCTAACTTCGTGCCAGCA
GCCGCGGTAATACGAAGGGTGCAAGCGTTAATCGGAATTACTGGGCGTAAAGCGTGCGCAGGCGGTTATGTAAGACAGTTGT
GAAATCCCCGGGCTCAACCTGGGAACTGCATCTGTGACTGCATAGCTAGAGTACGGTAGAGGGGGATGGAATTCCGCGTGTA
GCAGTGAAATGCGTAGATATGCGGAGGAACACCGATGGCGAAGGCAATCCCCTGGACCTGTACTGACGCTCATGCACGAAAG
CGTGGGGAGCAAACA

>ASV60
TGGGGAATATTGGACAATGGGCGAAAGCCTGATCCAGCCATGCCGCGTGTGTGAAGAAGGTCTTCGGATCGTAAAGCTCTGTT
GTTAGGGAAGAACAAATGTGTAAGTAACTGTGTACATCTTGACGGTACCTAACCAGAAAGCCACGGCTAACTACGTGCCAGCA
GCCGCGGTAATACGTAGGTGGCAAGCGTTATCCGGAATTATTGGGCGTAAAGCGCGCGTAGGCGGTTTTTTAAGTCTGATGTG
AAAGCCCACGGCTCAACCGTGGAGGGTCATTGGAAACTGGAAAACTTGAGTGCAGAAGAGGAAAGTGGAATTCCATGTGTAG
CGGTGAAATGCGCAGAGATATGGAGGAACACCAGTGGCGAAGGCGACCACCTGGACTGATACTGACACTGAGGTGCGAAAGC
GTGGGGAGCAAACA

>ASV61
TGGGGAATATTGGACAATGGGCGAAAGCCTGATGCAGCCATGCCGCGTGTATGAAGAAGGCCTTCGGGTTGTAAAGTACTTTC
AGTGGGGAGGAAGGCGATGAAGTTAATAACTTCGTCGATTGACGTTACCCGCAGAAGAAGCACCGGCTAACTTCGTGCCAGC
AGCCGCGGTAATACGAAGGGTGCAAGCGTTAATCGGAATTACTGGGCGTAAAGCGCGCGTAGGTGGTTCAGCAAGTTGGATG
TGAAATCCCCGGGCTCAACCTGGGAACTGCATCCAAAACTACTGAGCTAGAGTACGGTAGAGGGTGGTGGAATTTCCTGTGTA
GCGGTGAAATGCGTAGATATAGGAAGGAACACCAGTGGCGAAGGCGACCACCTGGACTGATACTGACACTGAGGTGCGAAAG
CGTGGGGAGCAAACA

>ASV62
TGGGGAATATTGCACAATGGGCGAAAGCCTGATGCAGCAACGCCGCGTGAGGGATGACGGCCTTCGGGTTGTAAACCTCTTTT
GGTAGGGAAGAAGGGAGCTTCGGTTCTTGACGGTACCTGCAGAAAAAGCACCGGCTAACTACGTGCCAGCAGCCGCGGTAAT
ACGTAGGGTGCAAGCGTTGTCCGGAATTATTGGGCGTAAAGAGCTCGTAGGCGGTGAATCGGGTTGAAAGTGAAAGTCGCCA
AAAAGTGGCGGAATGCTCTCGAAACCAATTCACTTGAGTGAGACAGAGGAGAGTGGAATTTCGTGTGTAGGGGTGAAATCCGT
AGATCTACGAAGGAACGCCAAAAGCGAAGGCAGCTCTCTGGGTCCCTACCGACGCTGGGGTGCGAAAGCATGGGGAGCGAAC
A

>ASV63
TAGGGAATCTTCCGCAATGGGCGAAAGCCTGACGGAGCAACGCCGCGTGAGTGATGAAGGTCTTCGGATCGTAAAGCTCTGT
TGTTAGGGAAGAACAAATGTGTAAGTAACTGTGCACATCTTGACGGTACCTAACCAGAAAGCCACGGCTAACTACGTGCCAGC
AGCCGCGGTAATACGTAGGGTGCGAGCGTTAATCGGAATTACTGGGCGTAAAGCGGGCGCAGACGGTTACTTAAGTCAGATG
TGAAATCCCCGGGCTCAACCTGGGAACTGCGTTTGAAACTGGGTGGCTAGAGTATGTCAGAGGGGGGTAGAATTCCACGTGT
AGCAGTGAAATGCGTAGAGATGTGGAGGAACACCAGTGGCGAAGGCGGCTTTCTGGTCTGCAACTGACGCTGATGTGCGAAA
GCGTGGGGATCAAACA

>ASV64
TAGGGAATCTTCCGCAATGGGCGAAAGCCTGACGGAGCAACGCCGCGTGAGTGATGAAGGTCTTCGGATCGTAAAGCTCTGT
TGTTAGGGAAGAACAAATGTGTAAGTAACTGTGCACATCTTGACGGTACCTAACCAGAAAGCCACGGCTAACTACGTGCCAGC
AGCCGCGGTAATACGTAGGTGGCAAGCGTTATCCGGAATTATTGGGCGTAAAGCGCGCGTAGGCGGTTTTTTAAGTCTGATGT
GAAAGCCCACGGCTCAACCGTGGAGGGTCATTGGAAACTGGAAAACTTGAGTGCAGAAGAGGAAAGTGGAATTCCATGTGTA
GCGGTGAAATGCGCAGAGATGTGGAGGAATACCGATGGCGAAGGCAGCCCCCTGGGATAATACTGACGTTCATGCCCGAAAG
CGTGGGGATCAAACA

>ASV65
TACGGAATCTTCCGCAATGGACGAAAGTCTGACGGAGCAACGCCGCGTGAGTGATGAAGGTTTTCGGATCGTAAAACTCTGTT
GTTAGGGAAGAACAAGTGCCGTTCGAAAGGGCGGCACCTTGACGGTACCTAACGAGAAAGCCACGGCTAACTACGTGCCAGC
AGCCGCGGTAATACGTAGGTGGCAAGCGTTGTCCGGAATTATTGGGCGTAAAGCGCGCGCAGGCGGTCTCTTAAGTCTGATGT
GAAAGCCCCCGGCTCAACCGGGGAGGGTCATTGGAAACTGGGAGACTTGAGTACAGAAGAGGAGAGTGGAATTCCACGTGTA

GCGGTGAAATGCGTAGAGATGTGGAGGAACACCAGTGGCGAAGGCGACTCTCTGGTCTGTAACTGACGCTGAGGCGCGAAAG
CGTGGGGAGCAAACA

>ASV66
TGGGGAATATTGCGCAATGGGGGGAACCCTGACGCAGCCATGCCGCGTGAATGAAGAAGGCCTTCGGATCGTAAAGCTCTGT
TGTTAGGGAAGAACAAGGATGAGAGTAACTGTTCATCCCTTGACGGTATCTAACCAGAAAGCCACGGCTAACTACGTGCCAGC
AGCCGCGGTAATACGTAGGTGGCAAGCGTTGTCCGGATTTATTGGGCGTAAAGCGAGCGCAGGCGGTTTTTTAAGTCTGATGT
GAAAGCCCACGGCTCAACCGTGGAGGGTCATTGGAAACTGGAAAACTTGAGTGCAGAAGAGGAAAGTGGAATTCCATGTGTA
GCGGTGAAATGCGCAGAGATATGGAGGAACACCAGTGGCGAAGGCGGCTTTCTGGTCTGCAACTGACGCTGATGTGCGAAAG
CGTGGGGATCAAACA

>ASV67
TGGGGAATTTTGGACAATGGGCGCAAGCCTGATCCAGCCATGCCGCGTGTCTGAAGAAGGCCTTCGGGTTGTAAAGGACTTTT
GTCAGGGAAGAAAAGCTTGAGGCTAATACCCTTGAGTGATGACGGTACCTGAAGAATAAGCACCGGCTAACTACGTGCCAGC
AGCCGCGGTAATACGTAGGTGGCAAGCGTTATCCGGAATTATTGGGCGTAAAGCGCGCGTAGGCGGTTTTTTAAGTCTGATGT
GAAAGCCCACGGCTCAACCGTGGAGGGTCATTGGAAACTGGAAAACTTGAGTGCAGAAGAGGAAAGTGGAATTCCATGTGTA
GCGGTGAAATGCGCAGAGATATGGAGGAACACCAGTGGCGAAGGCGGCTTTCTGGTCTGCAACTGACGCTGATGTGCGAAAG
CGTGGGGATCAAACA

>ASV68
TGGGGAATATTGGACAATGGGCGAAAGCCTGATCCAGCCATGCCGCGTGTGTGAAGAAGGCCTTCGGGTTGTAAAGTTCTTTC
GGTGATGAGGAAGGGGTATTATTGAATAGATAATATCATTGACGTTAATTACAGAAGAAGCACCGGCTAACTCCGTGCCAGCA
GCCGCGGTAATACGGAGGGTGCGAGCGTTAATCGGAATAACTGGGCGTAAAGGGCACGCAGGCGGACTTTTAAGTGAGATGT
GAAATCCCCGAGCTTAACTTGGGAACTGCATTTCAGACTGGGAGTCTAGAGTACTTTAGGGAGGGGTAGAATTCCACGTGTAG
CGGTGAAATGCGTAGAGATGTGGAGGAATACCGAAGGCGAAGGCAGCCCCTTGGGAATGTACTGACGCTCATGTGCGAAAGC
GTGGGGAGCAAACA

>ASV69
TAGGGAATCTTCCGCAATGGGCGAAAGCCTGACGGAGCAACGCCGCGTGAGTGATGAAGGTCTTCGGATCGTAAAGCTCTGT
TGTTAGGGAAGAACAAATGTGTAAGTAACTGTGCACATCTTGACGGTACCTAACCAGAAAGCCACGGCTAACTACGTGCCAGC
AGCCGCGGTAATACAGAGGGTGCAAGCGTTAATCGGAATTACTGGGCGTAAAGCGCGCGTAGGTGGTTTGTTAAGTTGGATGT
GAAATCCCCGGGCTCAACCTGGGAACTGCATCCAAAACTGGCAAGCTAGAGTATGGTAGAGGGTGGTGGAATTTCCTGTGTA
GCGGTGAAATGCGTAGATATAGGAAGGAACACCAGTGGCGAAGGCGACCACCTGGACTGATACTGACACTGAGGTGCGAAAG
CGTGGGGAGCAAACA

>ASV70
TGGGGAATCTTGGACAATGGGCGAAAGCCCGATCCAGCAATATCGCGTGAGTGAAGAAGGGCAATGCCGCTTGTAAAGCTCT
TTCGTCGAGTGCGCGATCATGACAGGACTCGAGGAAGAAGCCCCGGCGAACTCCGTGCCAGCAGCCGCGGTAAGACGGGGGG
GGCAAGTGTTCTTCGGAATGACTGGGCGTAAAGGGCACGTAGGCGGTGAATCGGGTTGAAAGTGAAAGTCGCCAAAAACTGG
TGGAATGCTCTCGAAACCAATTCACTTGAGTGAGACAGAGGAGAGTGGAATTTCGTGTGTAGGGGTGAAATCCGGAGATCTAC
GAAGGAACGCCAAAAGCGAAGGCAGCTCTCTGGGTCCCTACCGACGCTGGAGTGCGAAAGCATGGGGAGCGAACG

>ASV71
TGGGGAATATTGGACAATGGGCGAAAGCCTGATCCAGCAATGCCGCGTGAGTGATGAAGGCCTTAGGGTTGTAAAGCTCTTTT
ACTCGGGATGATAATGACAGTACCGGGAGAATAAGCTCCGGCTAACTTCGTGCCAGCAGCCGCGGTAATACGAAGGGGGCTA
GCGTTGCTCGGAATTACTGGGCGTAAAGGGAGCGTAGGCGGACATTTAAGTCAGGGGTGAAATCCCGGGGCTCAACCTCGGA
ATTGCCTTTGATACTGGGTGTCTTGAGTATGAGAGAGGTATGTGGAACTCCGAGTGTAGAGGTGAAATTCGTAGATATTCGGA
AGAACACCAGTGGCGAAGGCGACATACTGGCTCATTACTGACGCTGAGGCTCGAAAGCGTGGGGAGCAAACA

>ASV72
TGGGGAATATTGCACAATGGGCGCAAGCCTGATGCAGCGACGCCGCGTGGGGGATGACGGCCTTCGGGTTGTAAACCTCTTT
CAGCAGGGACGAAGCGCAAGTGACGGTACCTGCAGAAGAAGCACCGGCCAACTACGTGCCAGCAGCCGCGGTAATACGTAGG
GTGCGAGCGTTGTCCGGAATTACTGGGCGTAAAGAGCTCGTAGGTGGTTTGTCGCGTTGTTCGTGAAAACCGGGGGCTTAACC
CTCGGCGTGCGGGCGATACGGGCAGACTGGAGTACTGCAGGGGAGACTGGAATTCTTGGTGTAGCGGTGGAATGCGCAGATA
TCAGGAGGAACACCGGTGGCGAAGGCGACCACCTGGACTGATACTGACACTGAGGTGCGAAAGCGTGGGGAGCAAACA

>ASV73

TAGGGAATCTTCCACAATGGACGCAAGTCTGATGGAGCAACGCCGCGTGAGTGAAGAAGGCTTTCGGGTCGTAAAACTCTGTT
GTTGGAGAAGAACACGTTTGAGAGTAACTGTTCAGACGTTGACGGTATCCAACCAGAAAGCCACGGCTAACTACGTGCCAGCA
GCCGCGGTAATACGTAGGTGGCAAGCGTTATCCGGATTTATTGGGCGTAAAGCGAGCGCAGGCGGTTTTTTAAGTCTGATGTG
AAAGCCCTCGGCTTAACCGAGGAAGTGCATCGGAAACTGGGAAACTTGAGTGCAGAAGAGGACAGTGGAACTCCATGTGTAG
CGGTGAAATGCGTAGATATATGGAAGAACACCAGTGGCGAAGGCGGCTGTCTGGTCTGTAACTGACGCTGAGGCTCGAAAGC
ATGGGTAGCGAACA

>ASV74
TGGGGAATATTGCACAATGGGCGCAAGCCTGATGCAGCCATGCCGCGTGTGTGAAGAAGGCCTTCGGGTTGTAAAGCACTTTC
AGCGGGGAGGAAGGCGGTGAGGTTAATAACCTCATCGATTGACGTTACCCGCAGAAGAAGCACCGGCTAACTCCGTGCCAGC
AGCCGCGGTAATACGTAGGTGGCAAGCGTTATCCGGAATTATTGGGCGTAAAGCGCGCGCAGGTGGTTTCTTAAGTCTGATGT
GAAAGCCCACGGCTCAACCGTGGAGGGTCATTGGAAACTGGGAGACTTGAGTGCAGAAGAGGAAAGTGGAATTCCATGTGTA
GCGGTGAAATGCGTAGAGATATGGAGGAACACCAGTGGCGAAGGCGACTTTCTGGTCTGTAACTGACACTGAGGCGCGAAAG
CGTGGGGAGCAAACA

>ASV75
TAGGGAATCTTCCGCAATGGACGAAAGTCTGACGGAGCAACGCCGCGTGAGTGATGAAGGCTTTCGGTATGTAAAGCTCTATC
AGCAGGGAAGATAATGACGGTACCTGACTAAGAAGCCCCGGCTAACTACGTGCCAGCAGCCGCGGTAATACGTAGGTGGCAA
GCGTTATCCGGAATTATTGGGCGTAAAGCGCGCGCAGGTGGTTTCTTAAGTCTGATGTGAAAGCCCACGGCTCAACCGTGGAG
GGTCATTGGAAACTGGGAGACTTGAGTGCAGAAGAGGAAAGTGGAATTCCATGTGTAGCGGTGAAATGCGTAGAGATATGGA
GGAACACCAGTGGCGAAGGCGACTTTCTGGTCTGTAACTGACACTGAGGCGCGAAAGCGTGGGGAGCAAACA

>ASV76
TAGGGAATCTTCCGCAATGGGCGAAAGCCTGATCCAGCCATGCCGCGTGTGTGAAGAAGGTCTTCGGATTGTAAAGCACTTTA
AGTTGGGAGGAAGGGCAGTAAATTAATACTTTGCTGTTTTGACGTTACCGACAGAATAAGCACCGGCTAACTCTGTGCCAGCA
GCCGCGGTAATACAGAGGGTGCAAGCGTTAATCGGAATTACTGGGCGTAAAGCGCGTAGGTGGTTTGTTAAGTTGGATGTG
AAATCCCCGGGCTCAACCTGGGAACTGCATCCAAAACTGGCAAGCTAGAGTATGGTAGAGGGTGGTGGAATTTCCTGTGTAGC
GGTGAAATGCGTAGATATAGGAAGGAACACCAGTGGCGAAGGCGACCACCTGGACTGATACTGACACTGAGGTGCGAAAGCG
TGGGGAGCAAACA
